# RPA engages telomeric G-quadruplexes more effectively than CST

**DOI:** 10.1093/nar/gkad315

**Published:** 2023-05-04

**Authors:** Conner L Olson, Alexandra T Barbour, Thomas A Wieser, Deborah S Wuttke

**Affiliations:** Department of Biochemistry, University of Colorado Boulder, Boulder, CO80309, USA; Department of Biochemistry, University of Colorado Boulder, Boulder, CO80309, USA; Department of Biochemistry, University of Colorado Boulder, Boulder, CO80309, USA; Department of Biochemistry, University of Colorado Boulder, Boulder, CO80309, USA

## Abstract

G-quadruplexes (G4s) are a set of stable secondary structures that form within guanine-rich regions of single-stranded nucleic acids that pose challenges for DNA maintenance. The G-rich DNA sequence at telomeres has a propensity to form G4s of various topologies. The human protein complexes Replication Protein A (RPA) and CTC1-STN1-TEN1 (CST) are implicated in managing G4s at telomeres, leading to DNA unfolding and allowing telomere replication to proceed. Here, we use fluorescence anisotropy equilibrium binding measurements to determine the ability of these proteins to bind various telomeric G4s. We find that the ability of CST to specifically bind G-rich ssDNA is substantially inhibited by the presence of G4s. In contrast, RPA tightly binds telomeric G4s, showing negligible changes in affinity for G4 structure compared to linear ssDNAs. Using a mutagenesis strategy, we found that RPA DNA-binding domains work together for G4 binding, and simultaneous disruption of these domains reduces the affinity of RPA for G4 ssDNA. The relative inability of CST to disrupt G4s, combined with the greater cellular abundance of RPA, suggests that RPA could act as a primary protein complex responsible for resolving G4s at telomeres.

## Graphical Abstract

**Graphical Abstract:**
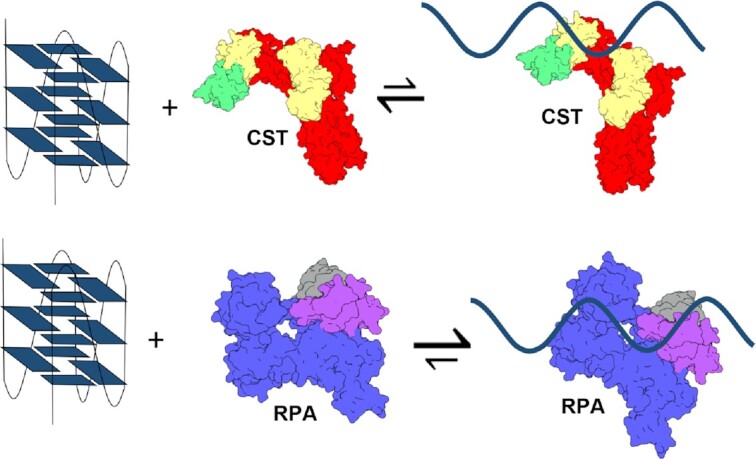
CST and RPA bind G-quadruplexes with differing propensities.

## INTRODUCTION

G-quadruplexes (G4s) are nucleic acid secondary structures that can form in regions of guanine-rich single-stranded DNA (ssDNA) and have important impact on cellular function (1–3). G4s can form under circumstances where duplex DNA is unwound or at sites in the genome, such as telomeres, that are dedicated ssDNA (2). While the full scope of G4 function is still being parsed out, it has been proposed that G4s could have both beneficial and detrimental consequences in the cell (2). One potential adversarial effect is G4s could become a barrier for DNA replication and ultimately lead to replication fork stalling and DNA damage if left unresolved (4–6). G4s triggering replication stress at telomeres is of particular interest, as the G-rich repeat sequence (TTAGGG) makes human telomeres particularly susceptible to forming G4s (7). These G4s need to be efficiently unfolded at telomeres to allow proper DNA replication and maintain telomere length homeostasis.

As a result of these challenges, cells have robust mechanisms to resolve G4s when DNA is single stranded during DNA replication and at telomeres. Helicases such as PIF1 and WRN have been shown to unfold G4s on the replicating lagging strand while BLM resolves leading strand G4s, and helicases such as RTEL1 and DHX36 specifically unfold RNA G4s (8–13). Furthermore, ssDNA-binding proteins that are rich in oligonucleotide/oligosaccharide-binding domains (OB-folds) are known to bind and unfold G4s without the need for helicase activity (14). Human replication Protein A (RPA) is a highly abundant ssDNA-binding protein that performs essential functions at sites of DNA replication, repair, and recombination (15–17). RPA has been shown to prevent the formation of G4s at telomeres, aid helicases in unfolding stable G4s and unfold G4s by itself (18,19). Human CTC1-STN1-TEN1 (CST) is an RPA-like protein complex that acts as a processivity factor to polymerase-alpha-primase at telomeres and provides genome stability at sites of DNA repair and replication (20–26). In contrast to RPA which acts ubiquitously throughout the genome, CST has an inherent biochemical preference for G-rich sites and is localized to G-rich sites in chromatin (27–29). CST has been shown to bind telomeric G4s *in vitro* and has been suggested to resolve G4s *in vivo* at replication forks to prevent replication fork stalling (30,31). Previous studies have shown that for both RPA and CST, binding of the G4 leads to unfolding of the ssDNA secondary structure (30–33). Alternatively, G4s can be resolved by conformational selection, or a passive mechanism, as done by protection of telomeres 1 (POT1) which binds quickly and tightly to the linear (unstructured) form of the G4 (34).

RPA and CST both have roles at non-telomeric sites of ssDNA. It has long been thought that RPA cannot beneficially bind to telomeres in the cell because RPA binding would lead to activation of DNA repair damage response pathways and ultimately chromosome end-to-end fusions (35,36). But RPA binds ssDNA non-specifically and with high affinity and has even been shown to localize to telomeres during replication (37,38). Therefore, G4s have been proposed as a mechanism for telomeric proteins like CST and POT1 to outcompete RPA for telomeric binding sites (30–32). As RPA is much more abundant in the cell compared to CST (39,40), in the absence of other mechanisms of localization, CST would need to bind to G4s with higher affinity to outcompete RPA.

In this study we performed a quantitative analysis of the ability of CST and RPA to bind and resolve G4s. First, we examined how CST and RPA bound to telomeric G4s of varying lengths using classical salt-dependence to modulate ssDNA secondary structure (6,41). While RPA does not preferentially bind to G4s of a particular topology, we tested to see if the same is true for CST (42). We used RPA DNA-binding mutants to test which domains of RPA are most important for G4 binding and unfolding to better understand what differentiates RPA activities from those of CST. Our results suggest that CST and RPA bind G4s with different efficiency, suggesting two different mechanisms of binding. RPA is much more efficient at binding telomeric G4s of all lengths. Together, these results provide insight into the mechanism of G4 resolution by both CST and RPA. RPA’s enhanced ability to bind and resolve G4s compared to CST provide a possible functional role for RPA at telomeres.

## MATERIALS AND METHODS

### Reagents

Unless stated otherwise, reagents were purchased from Sigma Aldrich (St. Louis, MO), Thermo Fisher Scientific (Waltham, MA) or New England Bio Labs (Ipswich, MA).

### Expression and purification of CST

Expression and purification of human CST was performed as previously described (43). Briefly, the Multi-Bac expression system was used to stoichiometrically co-express the three CST subunits, with CTC1 containing a 2xFLAG tag on the N-terminus and both STN1 and TEN1 containing an N-terminal 6xHis tag. Baculovirus was amplified to a titer of at least 4.0 × 10^8^ PFU/mL (measured by Expression Systems, USA) before infection. Two liters of *Trichoplusia ni* (Tni) cells (Expression Systems, USA) were grown to a cell density of 1.5–2.5 × 10^6^ cells/ml and were infected with an M.O.I. of 2. Cells were then grown for 67–68 h at 27°C at 130 rpm.

To obtain recombinant protein, cells were pelleted via centrifugation at 1500 × *g* for 30 min at 4°C, and cell pellets were then resuspended in 100 ml of lysis buffer (300 mM NaCl, 15 mM imidazole pH 8.0, 50 mM HEPES pH 7.4, 1 mM DTT and 1 EDTA-free protease inhibitor tablets (Roche) per 1L of cells.). Cells were lysed via sonication and cell lysate was then subject to high-speed centrifugation at 30 000 × *g* for 45 min at 4°C. Clarified cell lysate was then added to 5 ml of Ni-NTA agarose resin (Gold Bio, USA). Resin was pre-equilibrated with lysis buffer before addition of lysate. Lysate was equilibrated with resin at 4°C for at least 2 h under stirring conditions. Resin was washed three times with 50 ml of wash buffer (300 mM NaCl, 15 mM imidazole pH 8.0, 50 mM HEPES pH 7.4, 1 mM DTT) prior to elution with 50 ml of Ni-NTA elution buffer (300 mM NaCl, 300 mM Imidazole pH 8.0, 50 mM HEPES pH 7.4, 1 mM DTT). Ni-NTA elution was then added to 5 ml of Anti-FLAG resin (GenScript, USA) and allowed to equilibrate overnight on a rotator at 4°C. Resin was pre-equilibrated with Ni-NTA elution buffer prior to addition of Ni-NTA elution. Ni-NTA elution containing unbound protein was then allowed to flow through before beads were washed 3× with 15 ml of wash buffer. CST was then eluted using FLAG elution buffer (0.25 mg/ml 3xFLAG peptide (APExBIO, USA), 300 mM NaCl, 15 mM imidazole pH 8.0, 50 mM HEPES pH 7.4, 1 mM DTT). CST was concentrated to a final concentration of 20–70 μM with a 100K molecular weight cutoff spin column (Thermo Fisher Scientific, USA). Purity of eluted CST was confirmed via SDS-PAGE stained with AcquaStain Protein Gel Stain (Bulldog Bio, USA). Purified CST was stored in a 5% glycerol solution at -70°C after being snap frozen in liquid-nitrogen.

### Expression and purification of RPA

Recombinant RPA was expressed and purified as previously described with minor modifications (44). Briefly, RPA was expressed from a pET15b plasmid containing all three subunits, with RPA70 and RPA14 containing N-terminal 6xHis tags. Site-directed mutagenesis was performed with mutagenic primers to create mutant RPA constructs. All mutations were confirmed with commercial Sanger sequencing. Recombinant RPA was expressed in *E. coli* BL21 (DE3)-pLysS expression cells (Novagen) where cells were transformed with the plasmids via heat-shock. Four 1 l cultures were grown in 2xyt broth with 1 mg/ml ampicillin and 0.34 mg/ml chloramphenicol at 37°C for 3.5 h to an OD_600_ of 1.0. Cultures were then incubated on ice for 30 min before inducing RPA expression with 1 mM isopropyl-β-d-thiogalactopyranoside. Protein was expressed for 16–18 h at 18°C. Cells were harvested via centrifugation at 6000 × *g* for 15 min at 4°C. Cell pellets were collected in falcon tubes and then stored at −20°C.

Pellets were thawed and resuspended in lysis buffer (500 mM NaCl, 10 mM imidazole, 20 mM HEPES pH 7.5, 10 μM ZnCl_2_, 5 mM β-mercaptoethanol with one EDTA-free protease inhibitor tablet (Roche)) with 30 ml of lysis buffer used per 1 l of cells. Cells were lysed via sonication and lysate was then subjected to centrifugation 27 000 × *g* for 25 min at 4°C. Clarified lysate was then added to 12 ml of Ni-NTA agarose resin (Gold Bio, USA). Resin was pre-equilibrated with lysis buffer before addition of lysate. Lysate was allowed to incubate with resin for 1 h at 4°C. Lysate was then allowed to flow through before beads were washed 3× with increasing concentrations of imidazole (10 mM for wash one, 30 mM for wash two and 50mM for wash three). Protein was then eluted with elution buffer (500 mM NaCl, 300 mM imidazole, 20 mM HEPES pH 7.5, 10 uM ZnCl_2_, 5 mM β-mercaptoethanol with one EDTA-free protease inhibitor tablet (Roche)). Ni-NTA elution was then concentrated at 4°C to a final volume of 500–1000 ul in a 30K molecular weight cutoff spin column (Thermo Fisher Scientific, USA). Concentrated eluent was loaded onto HiLoad 16/600 Superdex 200 size exclusion column (GE Healthcare) and eluted with SEC buffer (100 mM NaCl, 20 mM HEPES pH 7.5, 10 μM ZnCl_2_, 0.2 M l-arginine, 5mM β-mercaptoethanol). RPA containing fractions were then collected and concentrated to 100–200 μM. Purity of RPA was confirmed via SDS-PAGE stained with AcquaStain Protein Gel Stain (Bulldog Bio, USA). Purified RPA was stored at −70°C after being snap frozen in liquid-nitrogen.

### Oligonucleotide design

All oligonucleotides were ordered from Integrated DNA Technologies (IDT, Coralville, IA). All oligonucleotides using in binding experiments were 5,6-carboxyfluorescein labeled by IDT. For sequences of all oligonucleotides used see Supplementary Table S1. Oligonucleotides were designed to match various length of telomeric G/C strands or to match previously known G4 forming sequences found in the human genome that vary in topology (34). For G4-forming oligonucleotides, G4 formation was confirmed via circular dichroism spectroscopy. Native gel analysis confirmed G4 structures were formed from intramolecular and not intermolecular interactions. See Results sections for each for further details.

### Fluorescence anisotropy binding assay for *K*_d,app_ determination

The fluorescence anisotropy (FA)-based binding assay was done as previously described (45). Briefly, each binding reaction (20 μL sample volume) contained 375 or 750 pM of fluorescently labeled oligonucleotide in binding buffer (200 mM salt, 20 mM HEPES pH 7.4, 1 mM DTT). Serial dilutions were performed in a 384 well plate (Cat No: 3575, Corning Inc., Corning, NY) and were set to have a starting protein concentration of 1000 or 2000 nM for CST and 1000, 2000 or 5000 nM for RPA reactions. Each experiment included a control well with the labeled oligonucleotide in binding buffer without protein. Oligonucleotides were heated for 10 min at 80°C and then slow cooled to 22–25°C for 1 h before being added to protein dilutions. CST binding reactions were allowed to equilibrate for 90 min at 22–25°C in the dark and RPA binding reactions were allowed to equilibrate for 30 min, except for reactions done in KCl which were found to need a longer equilibration time (excluding Tel18, Tel40 and Tel60) and were equilibrated for 180 min at 22–25°C in the dark as a longer equilibration time was needed to reach equilibrium see Supplementary Figure S1. RPA binding fluorescence intensity (parallel and perpendicular polarizations) of each reaction were measured using a ClarioStar Plus FP plate reader (BMG Labtech, Ortenberg, Germany). These values were used to calculate FA values for each concentration of protein (Eq. 1).


(1) $$\begin{equation*}Anisotropy\ = \frac{{\left( {Parallel - Perpendicular} \right)}}{{\left( {Parallel + 2*Perpendicular} \right)}}\end{equation*}$$

The anisotropy values were fit using the quadratic equation for the single site binding by non-linear least squares fitting (Eq. 2) in order to determine the apparent dissociation constant, *K*_d,app_,


(2) $$\begin{equation*}{F}_A = \ O + \frac{S}{{2\left[ L \right]}}\left( {\left( {{K}_{d,app.} + \left[ P \right] + \left[ L \right]} \right) - \sqrt {{{\left( {{K}_{d,app.} + \left[ P \right] + \left[ L \right]} \right)}}^2 - 4\left[ P \right]\left[ L \right]} } \right)\end{equation*}$$

where *O* is the minimal anisotropy, *S* is the difference between the minimal anisotropy and maximum anisotropy, *P* is the concentration of protein, and *L* is the concentration of oligonucleotide. Plots were then normalized to plot fraction bound. Averages calculated are the mean values from experiments and students two-tailed t-test were done to determine statistical differences between binding data sets. Standard error of the mean is reported in the data tables. For binding curves that did not reach completion *K*_d,app_ values are reported as lower limits.

### Circular dichroism to detect G4 formation

Oligonucleotides for circular dichroism (CD) measurements were diluted in CD buffer (10 mM Tris−HCl pH 7.4, 200 mM salt) to a concentration of 0.2 mg/ml. Oligonucleotides were heated at 80°C for 10 min and then cooled at 22–25°C for 1 h. CD spectra were acquired using a Chirascan Circular Dichroism Spectrometer (Applied Photophysics Ltd, USA) and 0.5 mm path length cuvettes. CD spectra were recorded at 22–25°C from 200–340 nm with a 0.5 nm step size, 2.0 nm bandwidth, and 0.5 s integration time. Cuvettes were rinsed 3x with MilliQ water, then rinsed 2× with pure ethanol before being dried with filtered air between samples. Periodically a MilliQ water blank was run between samples to ensure cuvettes were clean and not contaminated by previous sample. Buffer-only samples were run as blanks and were subtracted from CD and absorbance spectra oligonucleotide containing samples. CD data were normalized to molar circular dichroism (Δϵ) based on DNA strand concentration using Equation (3),


(3) $$\begin{equation*}\Delta \varepsilon \ = \frac{{\rm{\theta }}}{{32980*c*l}}\end{equation*}$$

where θ is the CD ellipticity in millidegrees (mdeg), *c* is the molar concentration (mol/l) of the oligonucleotide, and *l* is the path length in cm (46).

### Circular dichroism melt to determine thermal stability of G4 structures

Oligonucleotides for circular dichroism (CD) melt measurements were prepared as described in Circular dichroism to detect G4 formation section. CD spectra were acquired using a Chirascan Circular Dichroism Spectrometer (Applied Photophysics Ltd, USA) and 0.5 mm path length cuvettes. CD spectra were recorded from 200–340 nm with a 1.0 nm step size, 2.0 nm bandwidth, and 0.5 s integration time. Sample containing cuvettes were heated from a range within 20–95°C and CD readings were taken in 5°C increments. Buffer (CD buffer: 10 mM Tris–HCl 7.4, 200 mM of indicated salt) only samples were run as blanks and were subtracted from CD and absorbance spectra oligonucleotide containing samples. CD data were normalized to molar circular dichroism (Δϵ) based on DNA strand concentration using Equation (3). To determine *T*_m_, Δϵ for a wavelength of a characteristic peak for each oligonucleotide (see Supplementary Figure S2 for wavelength chosen for each oligonucleotide) was plotted against temperature and data were fit to a Boltzmann sigmoidal curve (Eq. 4) and *T*_m_ was determined as the half-way point between the two transitions (47,48).


(4) $$\begin{equation*}{\rm{\Delta \varepsilon \ }} = {\rm{\ }}\frac{{A2 + \left( {A1 - A2} \right)}}{{1 - {e}^{\frac{{T - {T}_m}}{{dx}}}}}\end{equation*}$$

where *A*1 is minimal Δϵ, *A*2 is the max Δϵ, *dx* is the slope of the transition, and *T* is the temperature in °C.

### Native PAGE gels for G4 molecularity determination

Oligonucleotides were diluted to 20 nM in CD buffer (10 mM Tris–HCl 7.4, 200 mM salt) and heated at 80°C for 10 min and then cooled at 22–25°C for 1 h. While oligonucleotides were cooling, a 10% 19:1 acrylamide:bis-acrylamide, 0.25× TBE gel containing 100 mM salt (NaCl, KCl or LiCl) was pre-run at 75 V for 30 min at 22–25°C using a 0.25× TBE running buffer containing 100 mM of the desired salt. Stock 20 nM oligonucleotide were diluted to 18 nM with 50% glycerol and this solution was mixed. 8 ul of the glycerol containing oligonucleotide solution was load onto the gel. The gel was then run at 75 V for 60 min at 22–25°C. Gels were imaged using FAM setting (473 nm excitation), as each oligonucleotide used is fluorescently labeled on the 5’ end as described in *Oligonucleotide design*, on Typhoon FLA 9500 imager (GE Healthcare).

### Fluorescence anisotropy binding assay for stoichiometry determination

Fluorescence anisotropy (FA) measurements of protein/DNA complexes formed at saturating ligand concentrations and titrated protein concentrations were performed to assess RPA protein to ligand stoichiometry. Oligonucleotides were labeled as described in *Oligonucleotide design*. All oligonucleotides were diluted in binding buffer (200 mM salt, 20 mM HEPES pH 7.4, 1 mM DTT) matching those used in *Fluorescence anisotropy binding assay for K_d,app_ determination*. Oligonucleotide concentrations were about 25 times the determined *K*_d,app_ so that the protein present is fully bound until stoichiometric excess is achieved. Oligonucleotide concentrations used for binding reactions with RPA in LiCl containing buffer: Tel22 25 nM, Tel30 25 nM, Tel40 25 nM, Tel60 25 nM. The oligonucleotide concentrations used for binding reactions with RPA in KCl reactions were: Tel22 60 nM, Tel 30 25 nM, Tel40 25 nM, Tel60 25 nM. Serial dilutions of RPA were performed in a 384-well plate (Cat. No.: 3575, Corning Inc., Corning, NY) in LiCl and KCl binding buffer (200 mM salt, 20 mM HEPES pH 7.4, 1 mM DTT). The oligonucleotides were heated and cooled to ensure proper folding prior to binding as described above. Binding reactions were equilibrated at room temperature for 60 min for reactions conducted in LiCl and 180 min for reactions conducted in KCl. Fluorescence intensity of each reaction was measured using a ClarioStar Plus FP plate reader. Anisotropy was then calculated using Equation (1). Linear fits were determined for the saturated (all available ligand is bound) and unsaturated region. The protein concentration needed for all the ligand to be bound was determined by finding the intercept of the saturated linear fit and unsaturated linear fit with Equation (5).


(5) $$\begin{equation*}\left[ {Protein\ needed\ for\ saturation} \right] = \frac{{b2 - b1}}{{m1 - m2}}\end{equation*}$$

For Equation (5), *b*1 is the *y*-intercept and *m*1 is the slope for the saturated region of the reaction while *b*2 is the *y*-intercept and *m*2 is the slope, both for the unsaturated region of the reaction (49). The ratio of protein concentration needed to saturate to ligand concentration in the reaction is the protein to ligand stoichiometry. All reactions were performed in triplicate and the mean of these values are reported. A two-tailed t-test was used to determine if there is a statistically significant difference between the stoichiometry of linear ligands versus G-quadruplex ligands.

### Electrophoretic mobility shift assay (EMSA)

The 5’ end of Tel22 was labeled with γ-^32^P-ATP as described by Lim *et al.* (43) and diluted in binding buffer (20mM HEPES–HCl pH 7.4, 200mM KCl) for a final concentration of 1 nM. For secondary structure folding, the oligonucleotide was heated at 80°C for 10 min, then slow cooled to 22–25°C for over 60 min. Serial dilutions of CST were made with a starting concentration of 1000 nM into binding buffer containing 50% glycerol. Binding reactions equilibrated at 22–25°C for 90 min. The reactions were then loaded onto a 0.7% agarose, 1× TBE gel and run with 1XTBE containing 100 mM KCl at 70 V at 4°C for 60 min. The gel was dried and the exposed on a Phosphoscreen for 18–24 h. The Phosphoscreen was imaged on a Typhoon FLA 9500 imager (GE Healthcare).

## RESULTS

### The ability of CST to bind telomeric DNA is inhibited by the presence of G4 structure

**Figure 1.**
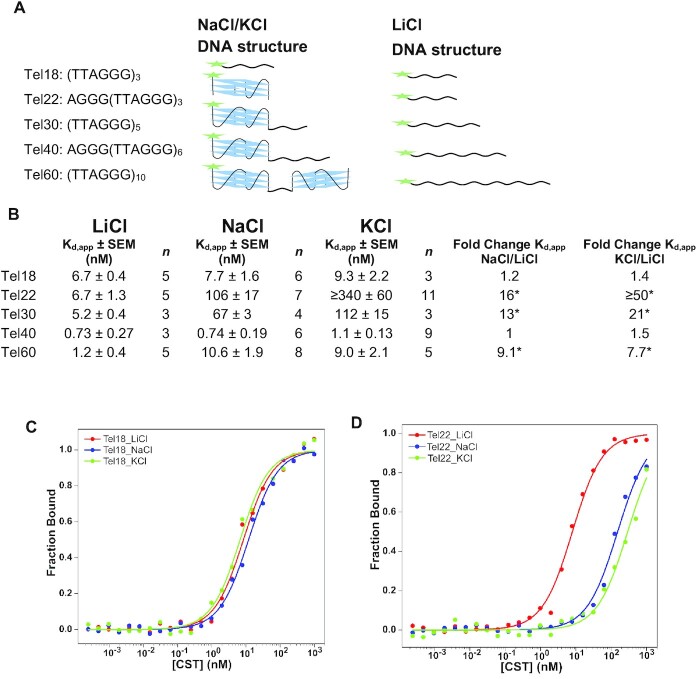
G4 structures inhibit ability of CST to bind telomere ssDNA. (**A**) Secondary structure of DNA oligonucleotides in each salt used for FA assays. Note for Tel30, Tel40 and Tel60 in NaCl and KCl there are multiple locations a G4 can form and consequently there will be a mixed population of structures. Structures shown here where the G4s are at the furthest 5’ end are for comparison and simplicity. Green star represents the FAM label. (**B**) CST’s binding affinites for telomere oligonucleotides used in FA assays in NaCl, KCl and LiCl. The equilibrium dissociation constants values are shown as a mean ± standard error of the mean (SEM) of *n* number of experiments. Fold change is calculated as the *K*_d,app_ NaCl/*K*_d,app_ LiCl for each given telomere oligonucleotide.* Indicates the differences in binding affinity between G4 and linear ssDNA is statistically significant with a *P*-value of ≤ 0.05 as determined by a student's two-tailed *t*-test. **(C**) Example FA binding curves for CST to Tel18 in KCl (green), NaCl (blue) and LiCl (red). (**D**) Example FA binding curves for CST to Tel22 in KCl (green), NaCl (blue) and LiCl (red).

Previous work using single molecule approaches has shown that CST can unfold G4s and bind the ssDNA in its extended conformation (30,31), but little is known about how CST’s binding ability is affected by various biologically relevant G4 structures that could form at telomeres. To test how G4s affect CST’s ability to bind telomeric ssDNA, all three CST subunits were co-expressed in insect cells and then purified via a double affinity pull down scheme which yielded pure CST (Supplementary Figure S2) (43). To directly compare CST’s binding to linear and G4 telomeric DNA, FA-based binding assays were done in buffers containing either NaCl or KCl, which favor G4 formation, or LiCl which disfavors G4 formation (Figure 1A) (6,41). The presence of DNA secondary structures, whether G4 (NaCl and KCl) or linear (LiCl), were confirmed using CD spectrometry (Supplementary Figure S3). Native gels of these oligonucleotides run in all three salts showed a single band for telomeric oligonucleotides running at or below bands to C-strand oligonucleotides of the same length, indicating the G4 structures were homogeneous and arise from intramolecular interactions rather than intermolecular interactions (Supplementary Figure S4).

Initial binding assays were done in LiCl to test the affinity of CST to linear telomeric sequences of various lengths before assessing how G4 formation affected CST’s affinity for each oligonucleotide. For linear telomeric ssDNA, CST’s affinity generally increased as the length of the ssDNA increased (Figure 1B, Supplementary Figure S5A and Supplementary Table S2). CST’s affinity increased 10-fold for the longer Tel40 and Tel60 oligonucleotides compared to the shorter Tel18 and Tel22 oligonucleotides. (Figure 1B, Supplementary Figure S5A, S5B, and Supplementary Table S2). This trend is consistent with other studies that show a similar length dependence of binding affinity (43,50). To control for possible differences in CST’s inherent binding ability between the salts, binding was performed with CST’s canonical binding minimal sequence, Tel18, which is too short to form G4s at all conditions (Figure 1B) (27). There was no statistically significant difference for CST’s affinity to Tel18 in LiCl (*K*_d,app_ 6.7 ± 0.4 nM) compared to in NaCl (7.7 ± 1.6 nM) as determined by a Student's two-tailed *t*-test (Figure 1B, C, and Supplementary Table S2). It should also be noted that our measured *K*_d,app_ for CST to Tel18 in NaCl is consistent with *K*_d,app_ values that were determined in Lim *et al.* by radiolabeled EMSAs (*K*_d,app_ 6.4 ± 1.1 nM), showing that the fluorescent label used in our assay does not affect binding (43).

As anticipated, CST bound Tel22 in LiCl with similar affinity as Tel18 in LiCl or NaCl with a *K*_d,app_ of ∼7 nM (Figure 1B). Unexpectedly, CST bound the G4 forming Tel22 in NaCl markedly weaker than linear Tel18, with a *K*_d,app_ of 106 nM (Figure 1B and D). With CST showing no cation preference for the Tel18 oligonucleotide, which is too short to form a G4 under any condition, CST’s drastic 13-fold reduction in affinity in NaCl compared to LiCl must be due to G4 formation and not the change in cation (Figure 1B and D). This roughly 10-fold loss in affinity was also observed with the Tel30 and Tel60 oligonucleotides (Figure 1B). The lone exception was the Tel40 oligonucleotide which CST showed no change in affinity for the linear or G4 form. This is most likely due to the Tel40 G4 population containing a long ssDNA tail due to it being too short to form multiple G4s (Figure 1A). Tel22, Tel30 and Tel60 will form G4s with shorter tails, which likely thwart CST’s ability to find a stretch of ssDNA that it can bind, leaving CST unable to bind the G4 with high efficiency. Within the population of the Tel40 G4s the exact position of the G4 will vary and as a result the length of the ssDNA tail will also vary. To determine if CST prefers the ssDNA tail to be in a specific location relative to the G4, we tested CST’s affinity against Tel40-based oligonucleotides that only allowed for the formation of the G4 at the 5’,3’ and in the middle of the Tel40 sequence due to placement of the G4s within the sequence (Supplementary Figure S6). We found that CST showed no preference for the position of the ssDNA and bound each oligonucleotide with similar affinity in NaCl and LiCl. These results match previous results from the literature that shows CST has no preference for the location of the ssDNA for a telomere overhang structure, binding 5’ and 3’ overhangs with the same affinity, and that CST can facilitate stable binding with ssDNA stretches as short as 10 nts (30). Furthermore, CST’s lack of preference for a G4 at the 5’ or 3’ of Tel40, which give rise to an 18 nt overhang, compared the G4 in the middle, which gives overhangs of 8 or 10 nts, suggest that CST is unfolding the G4 in the Tel40 oligonucleotides and not merely binding the long ssDNA tail as CST requires >10 nts to facilitate stable ssDNA binding (27).

G-rich sequences can adopt various G4 topologies, including a hybrid, parallel or anti-parallel G4 conformation (Supplementary Figure S7A) which have been suggested to serve different functions genome wide (51). To determine whether CST differentiates between these G4 topologies, we compared CST’s affinity for telomeric sequences in KCl, which forms a mixture of hybrid 1 and 2 topologies, to that in NaCl, which form anti-parallel structures (Figure 1B and Supplementary Figure S5C) (34). CST had three-fold weaker binding for Tel22 in KCl compared to NaCl and roughly two-fold weaker binding for Tel30 in KCl compared to NaCl (Supplementary Table S2). One possible explanation in the differences in binding could be the difference in thermodynamic stability, as G4s are more stable in KCl than NaCl (34,41,52) To better distinguish if CST discriminates between G4 topologies, we used oligonucleotides that fold into each of the possible G4 conformations in KCl, after first confirming their topology structures via CD (Supplementary Figures S7 and S8). Native gels showed a single band for five of the topology G4s with the only exception, 2M27, still being predominately unimolecular (Supplementary Figure S9). Hybrid G4s hindered CST binding the most dramatically, displaying at least 100-fold weaker affinity compared to their linear form (Supplementary Figure S7B and Supplementary Table S3). In contrast, CST binding was almost completely unaffected by parallel G4 oligonucleotides 2LBY and 2M27, binding linear and G4 with similar affinities. (Supplementary Figure S7B and Supplementary Table S3). Anti-parallel G4s showed a moderate ability to inhibit CST binding with linear-versus-G4 fold-change values of 18 and 36 for G4s 2KM3 and 6GZN, respectively (Supplementary Figure S7B).

In order to confirm that the differences in binding were due to the structural features of the G4 and not their relative thermal stability, CD melt assays were performed to measure the thermal stability of the G4 structures. There was no correlation between the thermal stability of the G4s and their ability to inhibit CST binding for both the telomeric and topology oligonucleotides (Supplementary Figures S10 and S11). Thus, any differences in binding to the various G4 oligonucleotides was not due to the relative stability of the G4 structure. Overall, these data support the notion that G4s hamper CST’s ability to bind telomeric ssDNA, and their impact is dependent on the length of the ssDNA tail. Furthermore, these data suggest that the inhibition of CST binding to G4s is sensitive to topology, which would be unique to CST compared to the ssDNA binding proteins RPA and POT1 which show no preference for G4 unfolding based on topology (34,42).

### G4 structures negate the selectivity of CST for telomeric DNA

Interestingly, unlike other telomeric proteins, CST is not specific for telomeric sequence alone, as it has been shown to systematically associate with G-rich ssDNA in many regions of the genome (27–29). Furthermore, previous studies have shown that, even for non-G-rich ssDNA, as the length of ssDNA increases CST’s binding ability likewise increases (29,50). Therefore, we wanted to assess how CST specificity for linear telomeric G-strand DNA of various lengths is affected by G4 formation.

To this purpose we chose the telomeric antisense C-strand as our non-preferred DNA sequence and tested CST binding to C-strand oligonucleotides of the same length as the telomeric oligonucleotides tested earlier via FA in NaCl (Table 1). As expected, CST showed little affinity to the short C-strand oligonucleotides (Table 1 and Supplementary Figure S12). In contrast, CST showed high affinity for the long C-strand oligonucleotides, C40 and C60, with a *K*_d,app_ of 18 nM (Table 1 and Supplementary Figure S12). These results match previous findings which show CST affinity increases drastically for C-strand oligonucleotides of greater than 30 nts in length with sequences of at least 40 nts having similar affinity to CST’s canonical binding oligonucleotide Tel18 (Table 1) (50). Overall, while CST’s specificity decreases for the longer oligonucleotides (≥30 nts) compared to shorter oligonucleotides, with a >60-fold higher affinity for Tel18 than for C18, CST is still roughly 20 times more selective for linear telomeric ssDNA between 30–60 nts than the C-strand oligonucleotides of the same length (Table 1). Yet, for G4 forming oligonucleotides without long tails, CST loses almost all specificity with <2-fold higher affinity for the telomeric G-strand oligonucleotides that form G4s than the C-Strand ssDNA of the same length (Table 1). It should be noted that the neutral pH of the binding buffer would prevent these C-strand oligos from forming i-motif secondary structures (53,54). Therefore, CST does maintain selectivity for telomeric G-strand ssDNA even as the length of ssDNA increases, but G4s negate this selectivity and make CST effectively non-specific for telomere G-strand sequences.

**Table 1.** CST’s binding affinity values to C-strand oligonucleotides as determined by FA assay. Binding for C-strand was done in NaCl. Equilibrium binding constants are given as a mean ± SEM for *n* number of experiments. Fold changes are calculated for the K_d,app_ C-Strand/K_d,app_ telomeric oligonucleotide of the same length in either LiCl (linear ssDNA) or NaCl (G4 favoring). *Indicates the differences in binding affinity between telomeric and C-strand ssDNA is statistically significant with a p-value of ≤0.05 as determined by a Student's two-tailed *t*-test

Oligonucleotide	*K*_d,app_ ± SEM (nM)	*n*	Fold change *K*_d,app_ C-strand/Tel in LiCl	Fold change *K*_*d*,app_ C-strand/Tel in NaCl
C18: (CCCTAA)_3_	≥440 ± 60	4	≥66*	≥57*
C22: (CCCTAA)CCCT_3_	184 ± 28	4	28*	1.7
C30: AAT(CCCTAAA)_4_CCC	87 ± 9	4	17*	1.3
C40: T(CCCTAA)_6_CCC	17 ± 4	4	23*	23*
C60: AAT(CCCTAA)_9_CCC	18.2 ± 2.8	3	16*	1.7

### RPA binds G4s with tighter affinity than CST

CST is often described as an RPA-like complex as both are heterotrimeric ssDNA binding protein complexes made up of OB-folds and they share structural homology with one another (43). RPA is the predominant ssDNA binding protein in the cell and plays essential roles in DNA replication and repair (15). RPA binding ssDNA can signal for the ATR DNA repair pathway and is detrimental for telomere maintenance, consequently there is a need for telomere proteins to outcompete RPA for telomeric ssDNA (55). It has been previously proposed that G4s are one mechanism that allows POT1 to outcompete RPA at telomeres (32). As both CST and RPA are proposed to help resolve stalled replication forks, if CST’s ability to resolve G4s is better than RPA that could help distinguish it as a telomere specific replication factor (15,21,32,56). Therefore, we wanted to directly compare how RPA binds telomeric G4s compared to CST.

We conducted FA binding assays with purified recombinant human RPA with the same five telomeric oligonucleotides as done with CST (Figure 1A and Supplementary Figure S13). For linear telomeric DNA, RPA showed a tight affinity ranging from 0.3 to 0.9 nM for the various ligands (Table 2 and Supplementary Figure S14). Due to the tight binding, we wanted to ensure that we were not in the titration range for our assay at the ligand concentration (750 pM) used and tested RPA binding Tel22 at half the concentration (375 pM) of oligonucleotide used for our initial analysis. We found no change in the fit binding affinity at the lower concentration of Tel22 (Supplementary Table S4); suggesting we were outside the titration range and our binding values are accurate for RPA binding in LiCl. Overall, these values are similar to previously reported affinities for RPA to linear ssDNA, including linear telomeric ssDNA with *K*_d,app_ ranging from 0.2–5 nM (57–59). Furthermore, Wieser & Wuttke displayed that the affinities are not influenced by the presence of the fluorophore, as they performed a competition assay to show that the fluorophore attached to the oligonucleotide does not affect binding affinity (59).

**Table 2.** RPA’s binding affinities to telomere oligonucleotides in each salt as determined by FA assay. Equilibrium binding constant values are given as a mean ± SEM for *n* number of experiments

	NaCl		KCl		LiCl	
Oligonucleotide	*K*_d,app_ ± SEM (nM)	*n*	*K*_d,app_ ± SEM (nM)	*n*	*K*_d,app_ ± SEM (nM)	*n*
Tel18	0.73 ± 0.05	3	0.50 ± 0.09	4	0.66 ± 0.14	5
Tel22	1.23 ± 0.19	6	1.99 ± 0.09	4	0.35 ± 0.05	14
Tel30	0.96 ± 0.21	6	0.81 ± 0.14	5	0.32 ± 0.06	5
Tel40	1.3 ± 0.4	4	1.1 ± 0.4	6	0.90 ± 0.19	7
Tel60	0.79 ± 0.22	4	0.69 ± 0.24	4	0.80 ± 0.26	3

**Figure 2.**
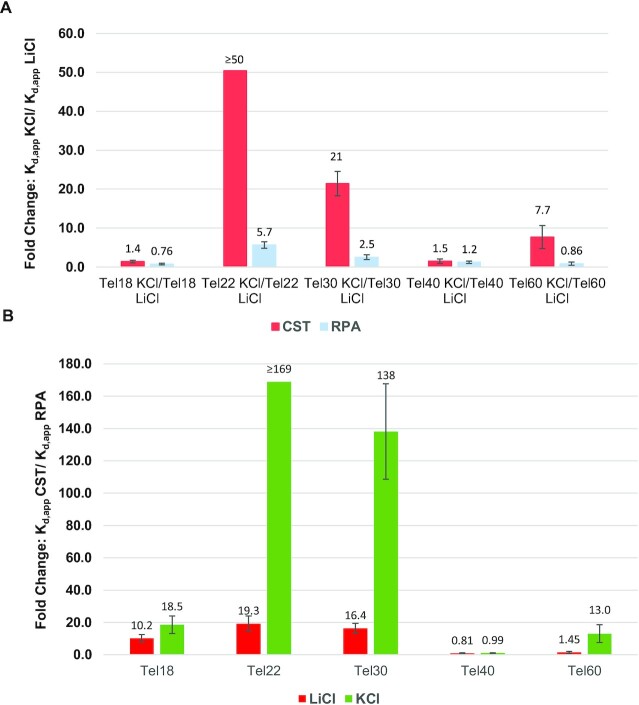
Telomere G4 structures inhibit CST binding ssDNA to a greater degree than RPA. (**A**) Comparison of how G4 formation directly affects CST and RPA binding. Fold changes are shown as *K*_d,app_ KCl/ *K*_d,app_ LiCl for each telomere oligonucleotide for CST (red) and RPA (blue). Where a larger fold change indicates the better the protein binds the oligonucleotide in LiCl than in KCl. (**B**) Comparison of how CST and RPA bind linear (LiCl) and G4 (KCl) telomere oligonucleotides. Fold changes are shown as *K*_d,app_ CST/*K*_d,app_ RPA for each telomere oligonucleotide for LiCl (red) and KCl (green). A larger fold change indicates the better RPA binds the oligonucleotide than CST. For both graphs error bars represent standard error of fold change. Binding to each oligonucleotide was performed *n*≥3 for both CST and RPA in each salt.

RPA, similar to CST, showed no difference in binding Tel18 in all three salts tested, meaning any differences in affinity for the longer oligonucleotides can be ascribed to G4 formation (Table 2 and Figure 2A). For both Tel40 and Tel60 RPA had no preference for ssDNA or G4, bound both equally robustly (Table 2 and Figure 2A). RPA had reduced affinity for Tel22 and Tel30 in the G4 form compared to the linear form, with a 5.7-fold lower affinity for Tel22 and 2.5-fold lower affinity for Tel30 in KCl compared to LiCl (Figure 2A). Yet, in both cases RPA’s binding was notably less impacted by the presence of G4 structures than CST’s (Figure 2B). The preference of RPA for the location of the ssDNA tail was also tested using the Tel40-based oligonucleotides that only allowed for the formation of the G4 at the 5’, 3’ and in the middle of the oligonucleotide. Like CST, RPA had no bias for the location of the ssDNA tail and bound all three structures without preference for ssDNA or G4 (Supplementary Table S5). Our results are consistent with literature which have shown RPA to have nanomolar affinity for telomeric G4s (32,60). Additionally, our data show that RPA has the same stoichiometry of protein:DNA in both KCl and LiCl for Tel22, Tel30, Tel40 and Tel60 (Supplementary Figure S15). This indicates that unfolding is occurring as RPA cannot stably bind G4 structures and there is not enough free ssDNA for each telomeric oligonucleotide for RPA to bind with the same stoichiometry as the linear ssDNA unless G4 unfolding occurred.

For both Tel22 and Tel30, CST’s binding affinity decreased 4-fold more in NaCl and 10-fold more in KCl than RPA compared to each protein's affinity in LiCl (Figure 2A and Supplementary Table S2). Binding to Tel60 by RPA was 10-fold less affected in both G4 stabilizing salts than observed for CST, as RPA showed similar binding affinity for telomeric ssDNA independent of G4 or linear secondary structure while CST’s affinity decreases 10-fold for G4s (Figure 2A and Supplementary Table S2). Thus, with Tel22, Tel30 and Tel60, CST’s ability to bind the ssDNA was more hindered by G4 formation compared to RPA. This conclusion can be further shown when directly comparing RPA’s and CST’s affinity to linear versus G4 telomeric ssDNA. RPA has a 15–20-fold higher affinity for the shorter telomeric sequences in their linear form (Figure 2B). RPA’s higher affinity for telomeric ssDNA is exacerbated when the oligonucleotides are G4s with RPA’s affinity over 160-fold tighter for Tel22 and 138-fold tighter for Tel30 than CST’s (Figure 2B). In both cases G4 formation increases RPA’s ability to outcompete CST for binding by >8-fold. This trend is also seen with the longer Tel60 (Figure 2B). CST binds the longer telomeric oligonucleotides Tel40 and Tel60 in their linear form with roughly the same affinity as RPA, while in the G4 form RPA binds Tel60 13-fold tighter than CST (Figure 2B). Tel40 again is the outlier as it does not hinder CST’s or RPA’s binding in the G4 state.

Overall, these data show that RPA binding to telomeric sequences is mostly unaffected by the presence of G4s and is much better at binding them compared to CST. These observations suggest that the presence of G4s would not favor CST binding and is not a viable mechanism to outcompete RPA for telomeric ssDNA. Rather, the existence of G4 structures in the target oligonucleotide would instead bias towards RPA binding.

### The DNA-binding domains of RPA work together to bind G4s

**Figure 3.**
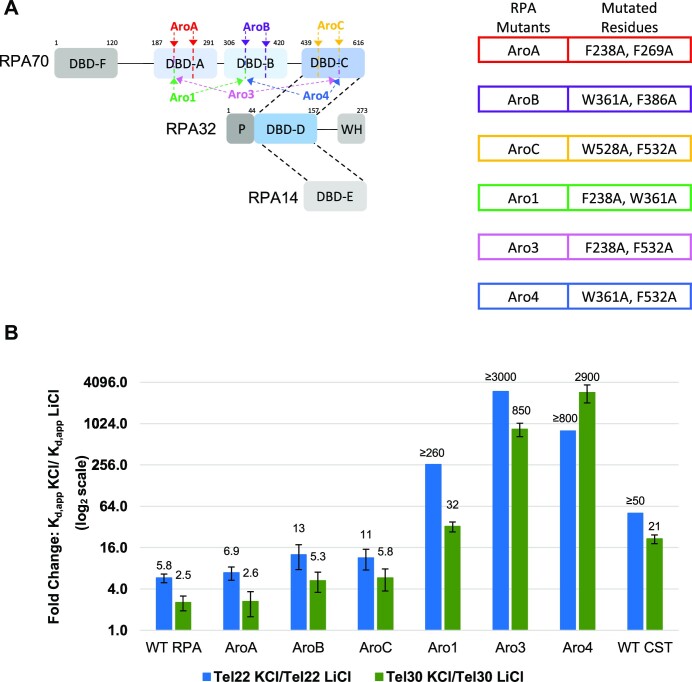
DNA binding domains of RPA work together to allow RPA to unfold G4s with greater efficiency than CST. (**A**) Domain map of RPA with mutated residues for each RPA mutant identified. Table listing specific mutations for each RPA mutant. **(B)** Fold changes for CST, WT RPA and mutant RPA’s to Tel22 LiCl compared to KCl (blue) and Tel30 LiCl compared to KCl (green). Fold changes are *K*_d,app_ KCl/*K*_d,app_ LiCl for each respective protein. Where a larger fold change indicates the better the protein binds the oligonucleotide in LiCl than in KCl. Fold change axis (y-axis) is shown in a log_2_ scale. Error bars represent standard error of fold change. Binding to each oligonucleotide was performed *n*≥ 3 for all proteins in both salts.

Recent work has shown that RPA binding is a highly dynamic process in which RPA uses its multiple DBDs to obtain various binding modes depending on the length of ssDNA and the DNA structure (57–59). Human RPA contains four ssDNA-binding OB folds, with three in the largest subunit, RPA70 and one in the medium subunit, RPA32 (37,57,61). CST contains at least three ssDNA-binding OB folds with at least two in the largest subunit, CTC1, and one in the medium subunit, STN1 (43,62). While both the smaller subunits of CST and RPA, STN1-TEN1 for CST and RPA32-RPA14 for RPA, share strong structural homology the larger DNA-binding subunits, CTC1 for CST and RPA70 for RPA, are less similar (43). For example, whereas structural overlays reveal that STN1 and RPA32’s are nearly identical, structural overlays for CST’s OB-E and RPA’s DBD-A show more limited homology (Supplementary Figure S16) (43). Consequently, we hypothesized that DBDs A and B differentiated RPA’s ability to bind G4s compared to CST. To test our hypothesis, we used known RPA mutants that would greatly diminish the binding ability of individual DBDs of RPA and tested their binding to the three shorter telomere oligonucleotides. This allowed us to identify the importance of the individual DBDs in mediating RPA’s ability to bind G4s (Figure 3A) (57,58,63). Previous structural and biochemical studies have shown that the aromatic residues F238 and F269 in DBD A, W361 and F386 in DBD B and W528 and F532 in DBD C are important for conferring interactions to short (>10 nt), long (∼30nt) and structured DNA substrates (59,64). Therefore, we mutated theses residues to alanine to test the importance of each DBD for binding G4s. Each mutant is named RPA AroX, with the X designating the DBD that contained the mutated residues (Figure 3A).

AroA, AroB and AroC RPAs all bound linear ssDNA with similar affinities to wild-type (WT) RPA (Table 3 and Supplementary Table S6). Correspondingly, all three mutants showed near WT like ability to bind telomeric G4s (Figure 3B). AroA mutant binding affinity was the least affected by telomeric G4 structures formed by Tel22 and Tel30 with a similar fold change to WT RPA (Table 2, Table 3 and Figure 3B). AroB and AroC were modestly affected, being roughly two times worse at binding G4 structures than WT RPA but was still roughly four or five times better at binding G4s than CST (Table 2, Table 3 and Figure 3B).These results support the emerging model that full-length RPA has multiple binding modes such that the decrease in affinity in one domain can be made up for by using a different binding mode (15).

**Table 3.** Binding affinites for mutant RPA’s to telomeric oligonucleotides as determined by FA assay. Equilibrium binding constant values for each protein are given as a mean ± SEM for *n* number of experiments

		AroA		AroB		AroC		Aro1		Aro3		Aro4	
		*K*_d,app_ ± SEM (nM)	*n*	*K*_d,app_ ± SEM (nM)	*n*	*K*_d,app_ ± SEM (nM)	*n*	*K*_d,app_ ± SEM (nM)	*n*	*K*_d,app_ ± SEM (nM)	*n*	*K*_d,app_ ± SEM (nM)	*n*
**Tel18**	NaCl	0.79 ± 0.12	4	0.50 ± 0.11	4	0.59 ± 0.14	6	4.7 ± 0.7	3	0.24 ± 0.03	4	3.5 ± 0.3	4
	KCl	0.34 ± 0.06	3	0.66 ± 0.18	5	0.26 ± 0.03	8	2.5 ± 0.3	6	0.56 ± 0.05	4	9.1 ± 0.3	3
	LiCl	0.36 ± 0.11	4	0.45 ± 0.15	4	0.49 ± 0.16	4	5.6 ± 1.4	4	0.65 ± 0.08	4	5.1 ± 0.5	4
**Tel22**	NaCl	2.4 ± 0.4	12	7.5 ± 1.1	6	2.3 ± 0.5	4	254 ± 24	8	26.1 ± 2.9	5	323 ± 19	5
	KCl	2.5 ± 0.3	7	6.7 ± 1.9	5	6.4 ± 1.0	4	>800	6	>2000	4	>5000	4
	LiCl	0.36 ± 0.06	9	0.53 ± 0.15	5	0.56 ± 0.16	4	3.1 ± 0.9	7	0.58 ± 0.07	6	6.9 ± 1.0	4
**Tel30**	NaCl	0.48 ± 0.12	13	1.07 ± 0.29	4	0.62 ± 0.11	5	8.2 ± 1.6	11	1.5 ± 0.4	5	15.0 ± 2.3	4
	KCl	0.77 ± 0.28	4	1.9 ± 0.3	3	1.62 ± 0.26	7	11.9 ± 2.8	5	175 ± 30	6	840 ± 90	4
	LiCl	0.29 ± 0.05	9	0.36 ± 0.07	4	0.28 ± 0.09	6	0.37 ± 0.04	3	0.21 ± 0.03	14	0.29 ± 0.08	4

DBDs A and B have been shown to act together to engage with ssDNA (15), so next we tested if mutating a single aromatic residue in two DBDs affected RPA’s ability to bind G4s. RPA with mutations in DBD A and B is called Aro1, mutations in DBD A and C together is called RPA Aro3, and mutations in DBD B and C together is called RPA Aro4 (Figure 3A). In contrast to mutating a single DBD, the Aro1 mutant showed roughly a ten-fold decrease in affinity for Tel18 and linear Tel22 in LiCl compared to WT but had WT like affinity for linear Tel30 (Supplementary Table S6). These results are consistent with prior studies that report that inhibition of the ability of DBDs A and B of RPA to bind ssDNA has the greatest impact on oligonucleotides shorter than 30 nts (58). Interestingly, this mutant displayed little ability to bind G4s (Figure 3B). For Tel30 Aro1 had a 22-fold decrease in binding affinity in NaCl and a 32-fold decrease in affinity in KCl (Figure 3B and Supplementary Figure S17). While for Tel22, Aro1 had an 82-fold decrease in affinity in NaCl and at least a 190-fold decrease in affinity in KCl (Figure 3B, and Supplementary Figure S17). Aro3 was able to maintain WT affinity to Tel18 and linear Tel22 and Tel30. However, unlike WT RPA, Aro3’s binding ability was greatly hindered by G4 formation. For Tel22 Aro3 had a 45-fold decrease in binding affinity in NaCl and >3000-fold reduction in affinity in KCl (Figure 3B). Its ability to bind the Tel30 was decreased 7.1-fold in NaCl and 850-fold in KCl (Figure 3B and Supplementary Figure S17). Aro4 was very similar to Aro1 in that its ability to bind Tel18 and linear Tel22 was affected, with an approximate 8-fold and 20-fold decrease in affinity compared to wild type, respectively (Supplementary Table S6). Yet, Aro4 had wild type like affinity for linear Tel30 (Supplementary Table S6). Similar to both Aro1 and Aro3, Aro4’s binding was greatly hampered by G4 formation (Figure 3B). Aro4’s ability to bind Tel30 was decreased 47-fold in NaCl and 2900-fold in KCl, while its binding ability was decreased 45-fold in NaCl and greater than 800-fold in KCl for Tel22 (Figure 3B and Supplementary Figure S17).

Taken together, RPA mutants Aro1, Aro3 and Aro4 are not only worse at engaging G4s than WT-RPA but their binding to shorter telomeric G4s is impacted by G4 formation more than CST (Figure 3B). These data support that multiple DBDs of RPA must be hindered for RPA’s binding ability to be extremely impeded by G4 formation. Furthermore, it supports that rather than just DBDs A and B of RPA working cooperatively together for binding, RPA can use different combinations of DBDs for multiple binding modes. Thus, if one DBD is unable to interact with the G4, it can pick another high affinity binding mode that can still bind G4s. However, inhibiting two of RPA’s DBDs greatly reduces the number of high-affinity binding modes RPA can use to bind the G4s, leaving RPA unable to efficiently bind them, reminiscent of the biochemical behavior of CST (59,64).

## DISCUSSION

### Insights into unfolding G4s by RPA versus CST

In this work, we show that telomeric G4s considerably inhibit CST’s ability to bind ssDNA thus substantially decreasing CST’s specificity for G-rich ssDNA. Furthermore, we show that RPA, the predominant ssDNA binding protein in the cell, is much more effective at binding G4s than CST. Moreover, RPA’s ability to bind G4s can be reduced by disrupting any combination of two DBDs of RPA70. The striking difference in the ability of CST and RPA to bind G4s, and the discovery that RPA binding affinity for G4s can be reduced through targeted mutagenesis, strongly suggests that the two protein complexes interact with and unfold G4s differently. We note that our observations differ than that reported by Zhang *et al.* (31). They reported CST binding to Tel24, which would form a G4 similar to Tel22, with an affinity of roughly 10 nM in 150 mM KCl, while we report an affinity to Tel22 of 340 nM in 200 mM KCl (31, Figure 1). We respectfully suggest a few differences in our studies could lead to these differing results. A potential contributor is the inherent differences in the method used to determine binding constants, Zhang *et al.* used electromobility mobility shift assays (EMSAs) while we used FA to measure binding. EMSAs are not an equilibrium binding technique, as they require the separation of bound and free species. Perturbing the equilibrium and allowing exchange back to other species would affect the *K*_d,app_. We used FA throughout this study which allows the equilibrium state to be directly measured thus avoiding the complications that may arise from needing to separate bound from unbound state. Furthermore, Zhang *et al.* did not include KCl in their running buffer which could destabilize G4 formation. In order to test if this contributed to the differences, we performed qualitative EMSAs at room temperature and included KCl in the running buffer. Qualitatively, we can see the *K*_d,app_ is similar to what we saw via FA, suggesting the presence of KCl in the running buffer helps maintain G4 structure (Supplementary Figure S18 and Figure 1).

Single molecule studies have shown that RPA and CST neither bind nor stabilize the G4, rather that binding is accompanied by G4 unfolding (30–33). Another OB-fold ssDNA-binding protein shown to unfold G4s is POT1 (32,34). POT1 is a component of the shelterin complex and robustly binds telomere ends (55). POT1 has been shown to unfold G4s through the conformational selection model where POT1 does not bind the G4 structure but captures the unfolded ssDNA that is in equilibrium with the folded G4 conformation (34,65). CST’s strong decrease in binding affinity to the G4 form of the ssDNA oligonucleotides is similar to that exhibited by POT1 (32,34). Furthermore, akin to POT1, CST’s ability to unfold G4s is hampered more in the presence of KCl than NaCl where the telomere G4 is more thermodynamically stable (Figures 1 and 2) (34,41,52). Kinetic experiments would be able to determine if CST unfolds G4s in a similar conformational selection mechanism as POT1 or uses a more active mechanism.

Our data further implies an additional mechanism CST can use to unfold G4s, based on the observation that CST has an increased ability to bind telomere G4s with longer ssDNA tails (Figures 1 and 2). Where the oligonucleotides that have ssDNA tails long enough for CST to bind (≥10 nts) independent of the G4 structure, CST can bind the available ssDNA which increases the local concentration of CST near the G4 and thus increasing its propensity to capture the unfolded state (Figure 1A). Additionally, binding of these tails could destabilize the G4 structure. Much the same as CST, POT1’s binding inhibition by G4s is somewhat alleviated by longer oligonucleotides which possess ssDNA stretches that POT1 can bind independent of G4 unfolding, suggesting POT1 can use this same mechanism (66).

Unlike both CST and POT1, RPA binding is mostly unaffected by telomere G4 formation (Table 2 and Figure 3). This suggests that RPA possesses a specific ability that makes G4 binding more effective. Previous studies have shown that RPA can melt double-stranded DNA if there is a stretch of ssDNA of just a few nucleotides long to initiate binding before the rest of the protein engages with more nucleotides (67) and this activity may extend to G4 structures as well. RPA’s ability to bind shorter segments of ssDNA, as small as 2–3 nts to facilitate stable binding, differentiates it from CST and POT1 which each need a longer stretch (>10 nts) for stable binding (27,68,69). Analysis of the cryo-EM structure of human CST indicates that OB-F interacts with about 3nts of ssDNA and OB-G interacts with about 6nts of ssDNA (62). RPA is known to adopt many binding modes (59,70) and structural information tells us the DBD-A interacts with about 3–4nts and DBD-B interacts with about 4nts (61). Although there are two important aromatic residues to confer RPA binding in DBDs A, B and C, we saw that mutagenesis of both residues in each DBD alone was not sufficient to disrupt RPA binding to G4s. However, when a single aromatic residue from two DBDs are mutated together, we see that RPA is no longer able to maintain tight binding of G4 structures. These data support the idea that multiple DBDs of RPA work together using various binding modes to bind and unfold G4s. This supplements the results presented by Prakash *et al.* that showed that each DBD of RPA plays a specific role for binding to ssDNA (71). They also concluded that DBDs C, D and E are important for binding and unfolding G-quadruplexes. Our data shows that additionally, DBDs A, B and C are crucial for binding G-quadruplexes. We believe differences in our results are due to the intrinsic differences when analyzing the full-length protein versus a domain analysis. Full-length RPA can exhibit plasticity when interacting with different ligands where the domains can work together to achieve affinity and specificity beyond that exhibited in isolation (59).

One possible model of RPA unfolding of G4s entails RPA initially binding the exposed ssDNA loops or tails of the G4, where it either destabilizes the G4 or is poised to capture the oligonucleotide as it samples the extended state. This mechanism is supported by our data where G4s with longer tails are more easily bound by RPA, and it consistent with previous work that shows G4 loop length affects ability to unfold G4s (Figure 3) (42). While active unfolding of G4s is usually associated with ATP-dependent helicases, DHX36 is a helicase which has been shown to unfold G4s in an ATP-independent manner using binding with an OB-fold, where the OB-fold tugs on the loops or tails of the G4, destabilizing the structure to reach the unfolded state (72). These results suggest a universal G4 unfolding mechanism for OB-fold containing ssDNA binding proteins where minimal nucleotide binding length of the protein and the length of the G4 loops and tail dictate whether the protein uses only conformational selection model, or if the protein can unfold the G4 in a more active manner if the minimal tail length is present.

### G4 resolution *in vivo*

The formation of G4s has also been suggested to play a role in helping telomere-specific proteins outcompete the much more abundant RPA for the telomere end (32). This is unlikely to apply to CST due to RPA’s greater efficiency at unfolding G4s. POT1’s association with shelterin via its interaction with TPP1 also helps it outcompete RPA, as it places POT1 in proximity to the telomeric ssDNA overhang, but there is no current evidence that CST can use the same mechanism (68,69). The question then is how CST outcompetes POT1 and RPA for the telomere end to initiate C-strand fill in.

CST helps initiate C-strand fill in by terminating the action of telomerase through competition for the ssDNA and recruiting pol α-primase to the telomere end where it acts as a processivity factor (20,45,73,74). Recent studies have shown CST’s ability to act as a processivity factor for pol α-primase goes beyond recruitment to the telomere end, as CST provides other benefits such as resolving DNA secondary structures like G4s (6,62). The biochemical data from Zaug *et al.* show that while CST increases pol α-primase's activity for G4 forming oligonucleotides, there was an accumulation of non-fully replicated products of roughly 32–40 nts (6). Interestingly, the non-G4 forming oligonucleotides tested had a population of longer, fully replicated products (6). These results support that CST captures the ssDNA, unwinding the G4 and allowing pol α-primase to begin replication. These results further suggest that, for longer stretches of telomere ssDNA which can form multiple G4s, CST cannot continue to unwind G4s beyond the initial ssDNA it binds. Consequently, pol α-primase is further inhibited and only allowed to replicate 30–40 nts at a time. This function would be independent of whether CST has ability to travel along ssDNA as pol α-primase continues to replicate. As RPA is also known to interact with pol α-primase, it will be interesting to understand how RPA functions with pol α-primase in comparison with CST.

Replication of the telomere presents challenges due to its G-rich nature. Both CST and RPA have been shown to play a role in saving stalled replication forks with one proposed mechanism being their ability to resolve DNA secondary structures and the concomitant recruitment of pol α-primase (15,22). Our work suggests that the available ssDNA from the G4 loops or tails will dictate whether RPA or CST would be effective at resolving the G4 barrier. Therefore, RPA will be able to efficiently resolve most G4s independent of a ssDNA tail flanking either side of the G4, while CST will need a long ssDNA stretch to bind to efficiently resolve a G4 structure. RPA is also known to interact with G4 resolving helicases, while CST on the other hand is not currently known to act with any G4 resolving helicases (15). This makes RPA is an ideal candidate to fulfill the role of rescuing forks from G4s as RPA can unfold the G4 itself or bind the ssDNA after a helicase acts (15). Interestingly, recent work has shown that RPA, not CST, is present during bulk DNA replication at telomeres (38). Altogether this supports the model where RPA is more suited to play a key role resolving G4s at telomeres and genome wide.

Due to their importance to genomic health and their overlapping structure and function, there is an increasing need to better understand the interplay between CST and RPA, both at telomeres and genome wide. Furthermore, due to CST’s significance in maintaining telomere health, and its recent interest as a cancer target (25,75), understanding the mechanistic details for where, when, and how CST competes with RPA action is crucial for understanding CST’s biological importance. Resolving DNA secondary structures is one such function both can provide for maintaining genomic stability. Further research is needed to better understand the signaling and regulation between these essential proteins and how their interactions with G4s in the cell impact DNA replication.

## DATA AVAILABILITY

All data are available from the corresponding authors upon reasonable request and/or included in the manuscript as figure source data or supplementary data.

## Supplementary Material

gkad315_Supplemental_File
