# Supplementary material for: RPA engages telomeric G-quadruplexes more effectively than CST
Source: Nucleic Acids Res. 2023 May 4;51(10):5073–86. doi: 10.1093/nar/gkad315 (PMC10250233; doi:10.1093/nar/gkad315)
Supplement: gkad315_Supplemental_File [file gkad315_supplemental_file.pdf]

Supplementary Data

RPA engages telomeric G-quadruplexes more effectively than CST

Conner L. Olson[1], Alexandra T. Barbour[1], Thomas A. Wieser[1], and Deborah S. Wuttke[1,*]

[1]Department of Biochemistry, University of Colorado Boulder, Boulder, CO 80309, USA,

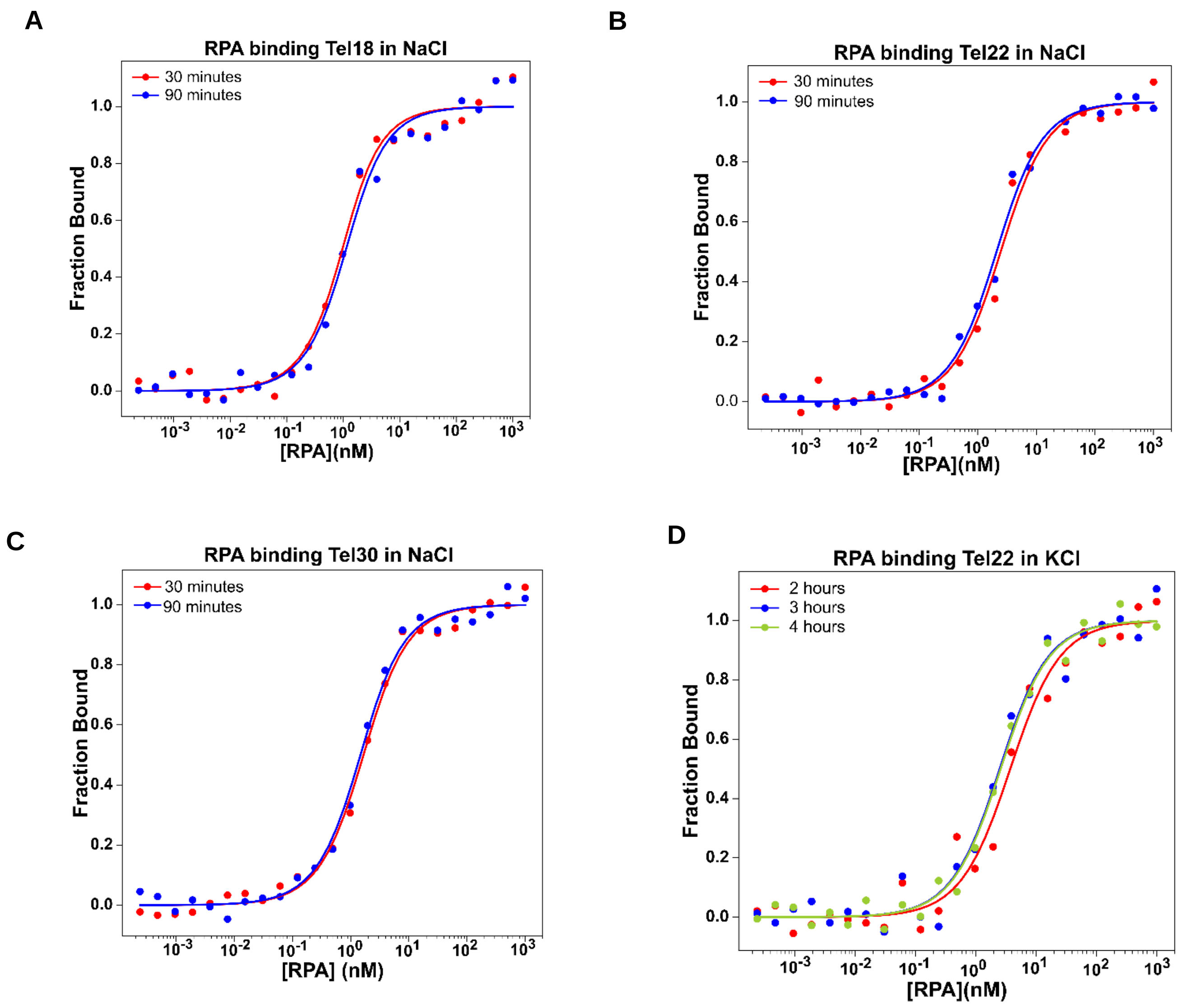

Supplementary Figure S1: RPA equilibration experiments. (A-C): Wild-type RPA binding Tel18 (A), and Tel22 (B), and Tel30 (C) in 200 mM NaCl at 30 and 90 minutes. These experiments show that equilibrium was reached after 30 minutes. This trend was seen with all oligos in all 3 salts with WT RPA except for Tel22 and Tel30 in 200 mM KCl. (D) WT RPA binding Tel22 in KCl at 2, 3 and 4 hours. These experiments show that equilibrium was reached after 3 hours. This was also seen with Tel30 in KCl. For all experiments oligonucleotide concentration was 0.75 nM.

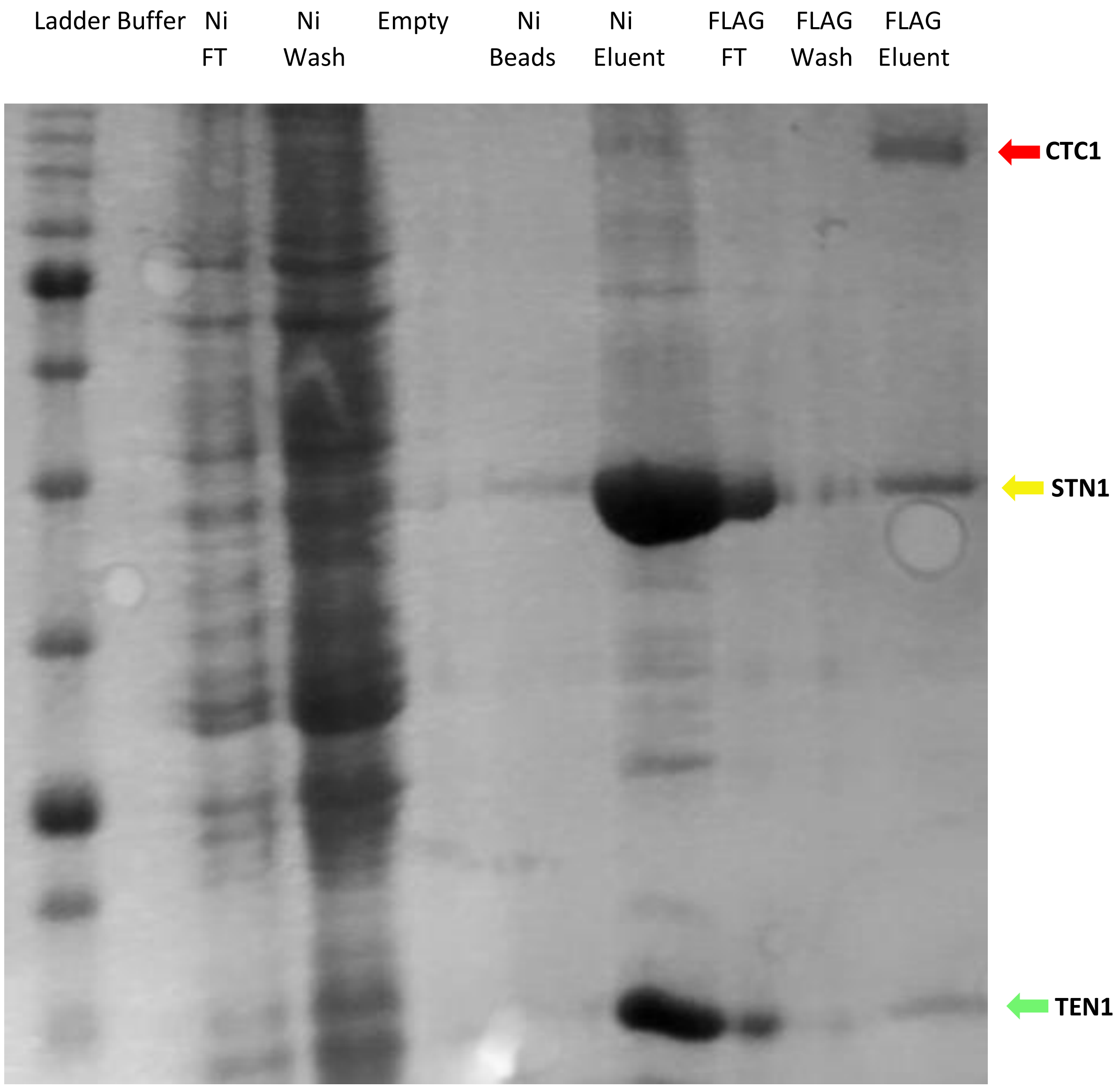

Supplementary Figure S2: CST purification gel. 12% SDS-PAGE gel for wild-type CST purification. In order on the gel: Protein ladder, buffer, Nickel Flow Through (FT), Nickel Wash, empty, Ni Beads Post elution, Nickel Eluent, Empty, FLAG flow through (FT), Flag wash, and Flag Eluent. CTC1 is indicated by the red arrow, STN1 is indicated by the yellow arrow and TEN1 is indicated by the green arrow.

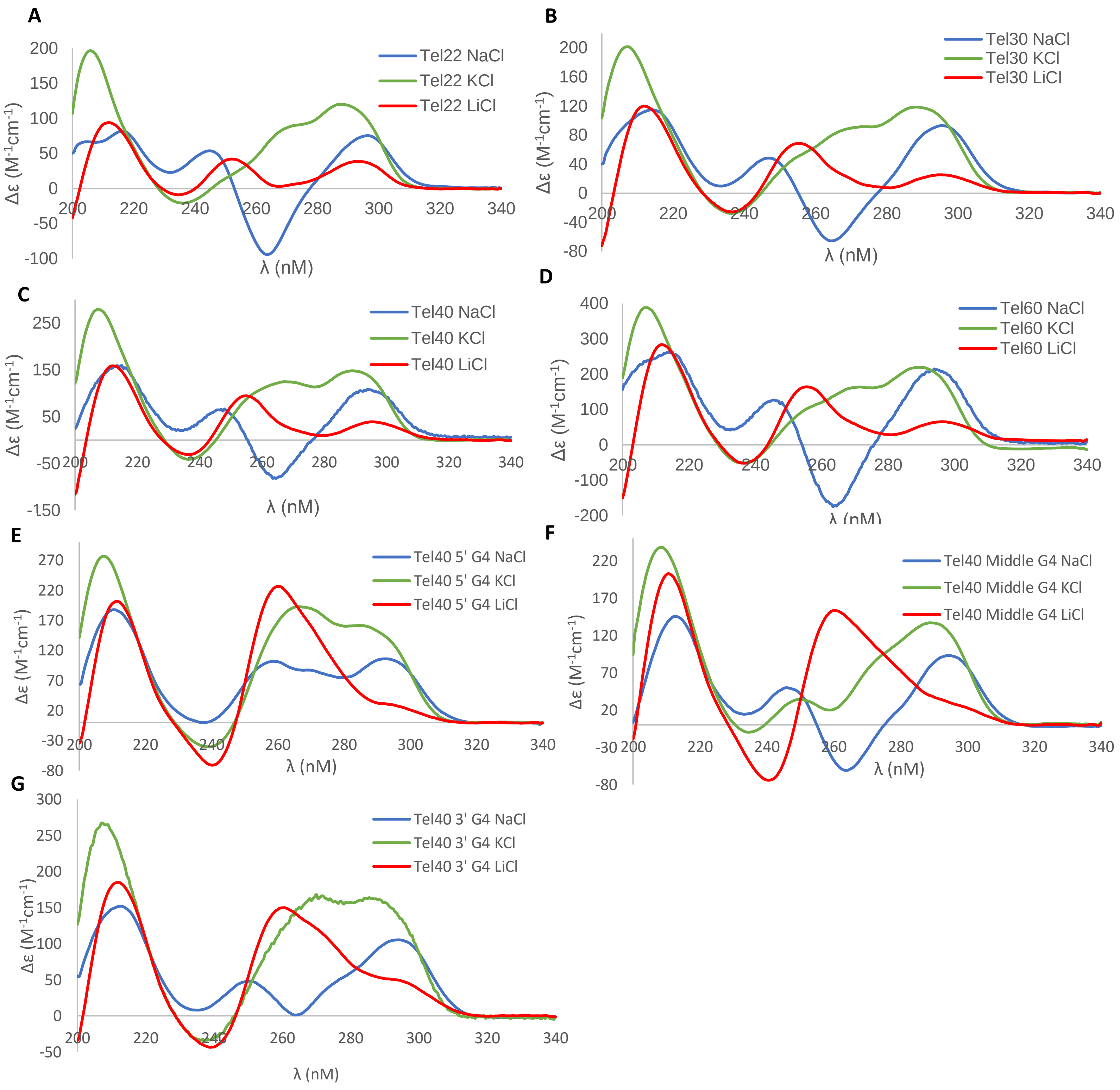

Supplementary Figure S3: Circular dichroism (CD) curves for telomeric oligonucleotides (A) CD spectrum for Tel22. (B) CD spectrum for Tel30. (C) CD measurements for Tel40. (D) CD spectrum for Tel60. (E) CD spectrum for Tel40 5' G4. (F) CD spectrum for Tel40 Middle G4. (G) CD spectrum for Tel40 3' G4. Measurements for G4 forming oligonucleotides in NaCl confirm an antiparallel topology a peak at ~290 nm and a trough at ~260 nm. Measurements for G4 forming oligos in KCl confirm hybrid topology with peaks at ~290 and ~260 nm and a trough at ~240 nm.

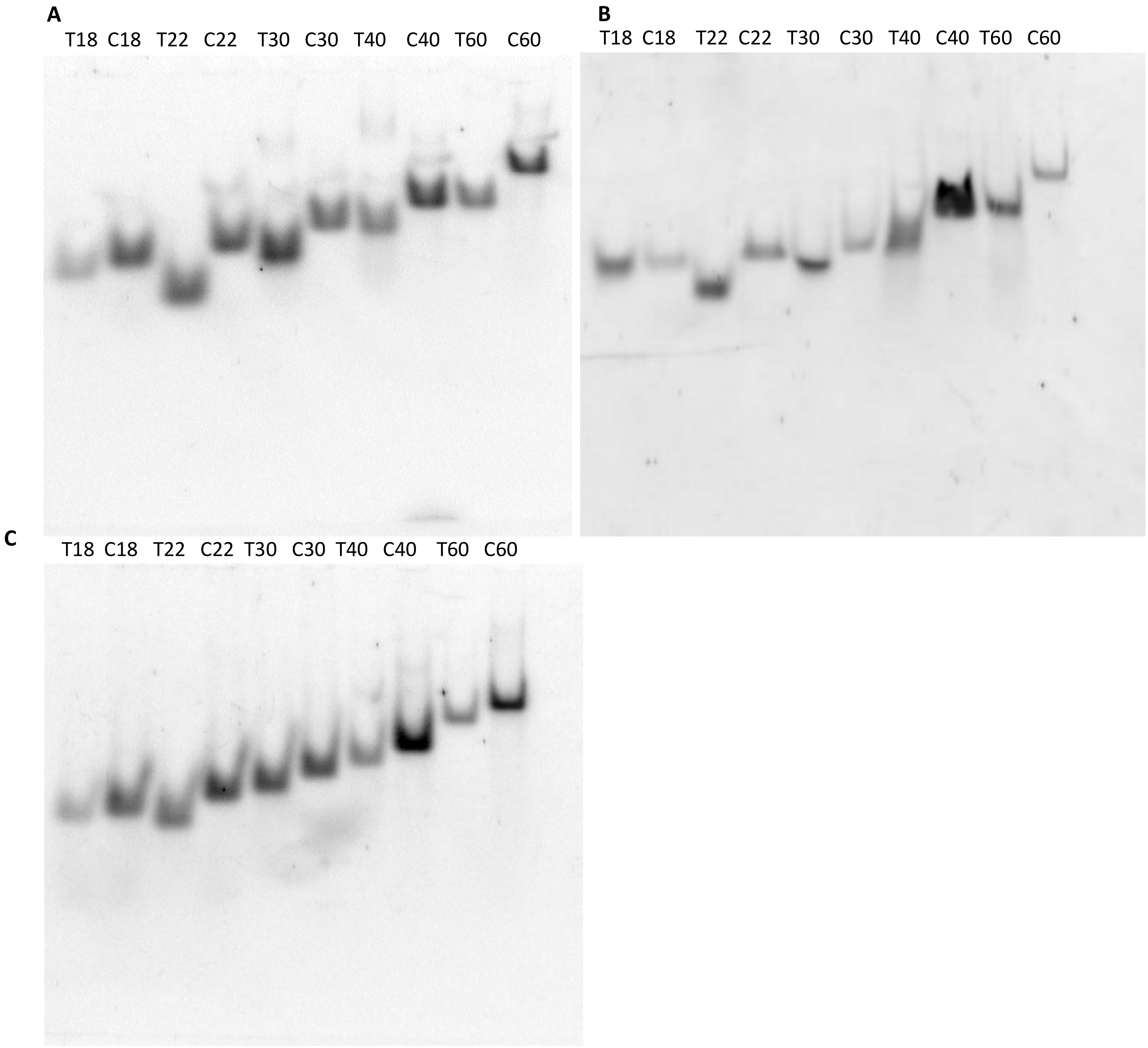

Supplementary Figure S4: Native PAGE gels confirming telomere G4s are unimolecular. (A) Native gel (0.25xTBE) in KCl (100 mM). Gel shows G4s are unimolecular for telomeric oligonucleotide. G4s travel further down the gel than the linear C-strand oligonucleotides with only one band present showing no intermolecular G4s formed. (B) Native gel (0.25xTBE) in NaCl (100 mM). Gel shows G4s are unimolecular for telomeric oligonucleotide. Similar to observations iKCl, G4s travel further down the gel than the linear C-strand oligonucleotides with only one band present showing no intermolecular G4s formed. (C) Native gel (0.25xTBE) in LiCl (100 mM). Gel shows linear telomeric oligonucleotides migrate similarly to the G4 telomeric oligonucleotides. In LiCl telomere oligonucleotides will be linear and therefore unimolecular. Telomere oligonucleotides migrate the same distance in KCl and NaCl as in LiCl with no second band present confirming that oligonucleotides are unimolecular in all three cases. Even in LiCl telomeric oligonucleotides have been shown to form compact unimolecular structures but not full G4s (1) explaining why the telomeric oligonucleotide still travels slightly further than the C-strand. For oligonucleotide labeling T18=Tel18 and C18=C-strand 18 nts.

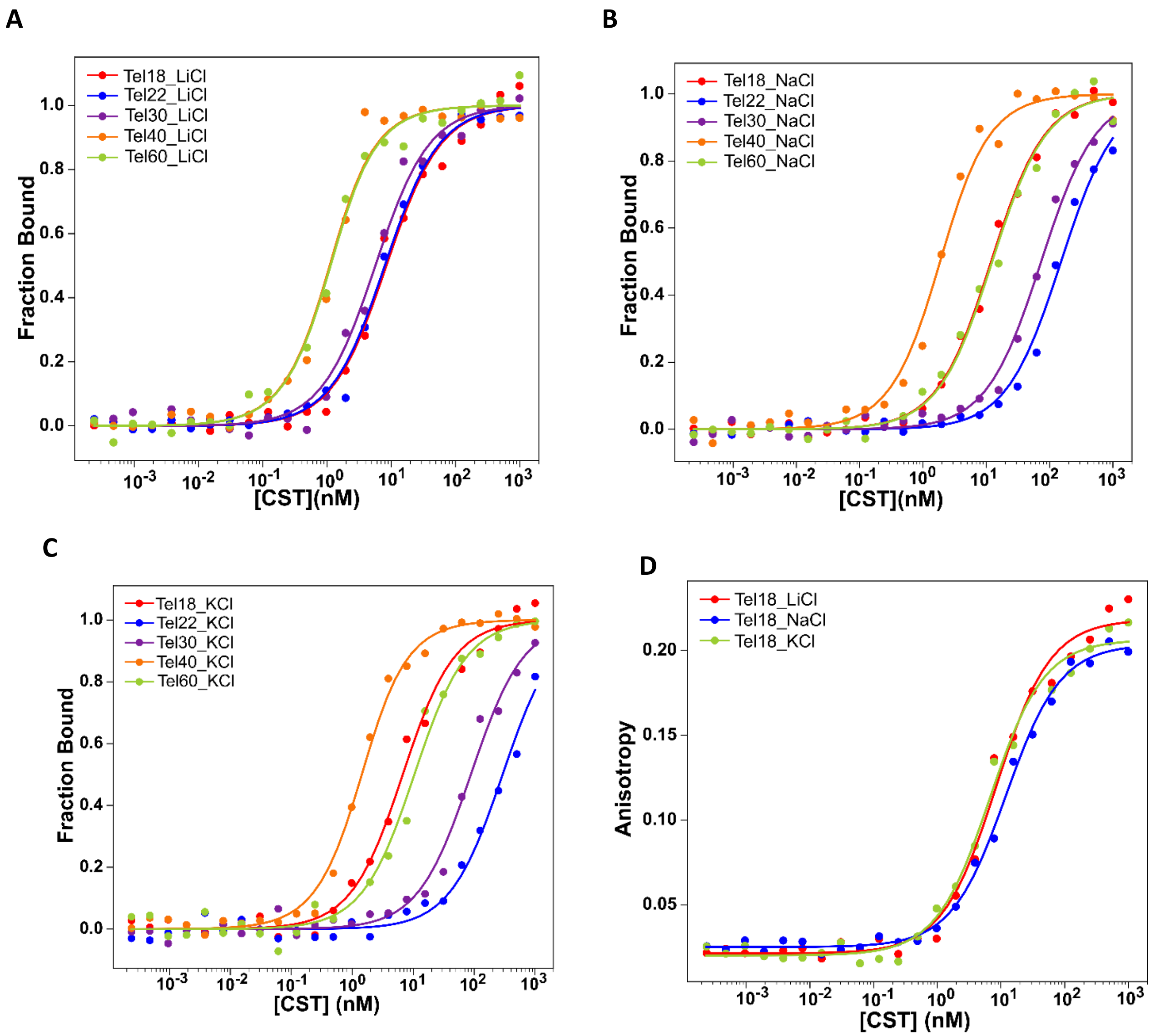

Supplementary Figure S5: (A) Example normalized FA binding curves for CST to telomere oligonucleotides in 200 mM LiCl. (B) Example normalized FA binding curves for CST to telomeric oligonucleotides in 200 mM NaCl. (C) Example normalized FA binding curves for CST to telomeric oligonucleotides in 200 mM KCl. Curves for both A, B and C were fit using equation 1. (D) Representative FA binding curves for CST to Tel18 showing range of anisotropy values for binding. For FA binding curves that did not reach completion $K_{d,app}$ values are approximations for those oligonucleotides.

A

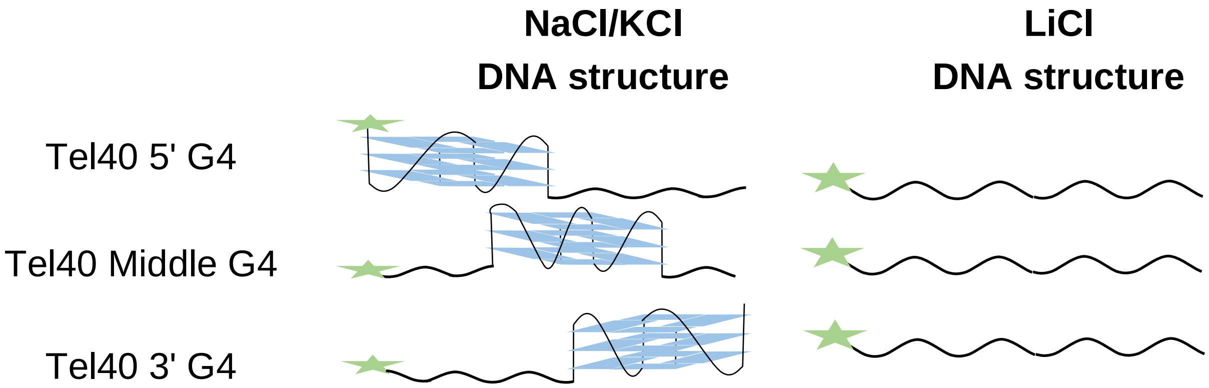

B

	LiCl $K_{d,app}$ ± SEM (nM)	*n*	NaCl $K_{d,app}$ ± SEM (nM)	*n*	KCl $K_{d,app}$ ± SEM (nM)	*n*	Fold Change $K_{d,app}$ NaCl/LiCl	Fold Change $K_{d,app}$ KCl/LiCl
Tel40	0.73 ± 0.27	3	0.74 ± 0.19	6	1.1 ± 0.13	9	1.0	1.5
Tel40 5' G4	1.53 ± 0.17	4	1.47 ± 0.23	4	6.2 ± 0.3	4	0.96	4.0*
Tel40 Middle G4	1.94 ± 0.16	4	1.04 ± 0.21	4	4.4 ± 0.3	4	0.53*	2.3*
Tel40 3' G4	1.09 ± 0.10	5	2.3 ± 0.4	4	3.4 ± 0.5	4	2.1*	3.1*

Supplementary Figure S6: The position of G4 does not alter CST's binding affinity for telomeric ssDNA. (A) Secondary structures of Tel40 G4 oligonucleotides in each salt used for FA assays (B) Binding affinites for wild-type CST to telomeric oligonucleotides of 40 nucleotides in length which vary where the G4 forms. Binding done in NaCl, KCl and LiCl. For fold change. *Indicates the differences in binding affinity between G4 and linear ssDNA is statistically significant with a p-value of ≤ 0.05 as determined by a student's two-tailed t-test.

A

DNA Ligand	Topology	Length	Sequence
2GKU	Hybrid	24nt	TT(GGGTTA)$_3$GGGA
2JSL	Hybrid	25nt	TA(GGGTTA)$_3$GGGTT
2KM3	Anti-Parallel	22nt	AGGG(CTAGGG)$_3$
6GZN	Anti-Parallel	20nt	GGGTAGGGAGCGGGAGAGGG
2LBY	Parallel	19nt	TAGGGAGGGTAGGGAGGGT
2M27	Parallel	22nt	CGGGGCGGGCCTTGGGCGGGGT

B

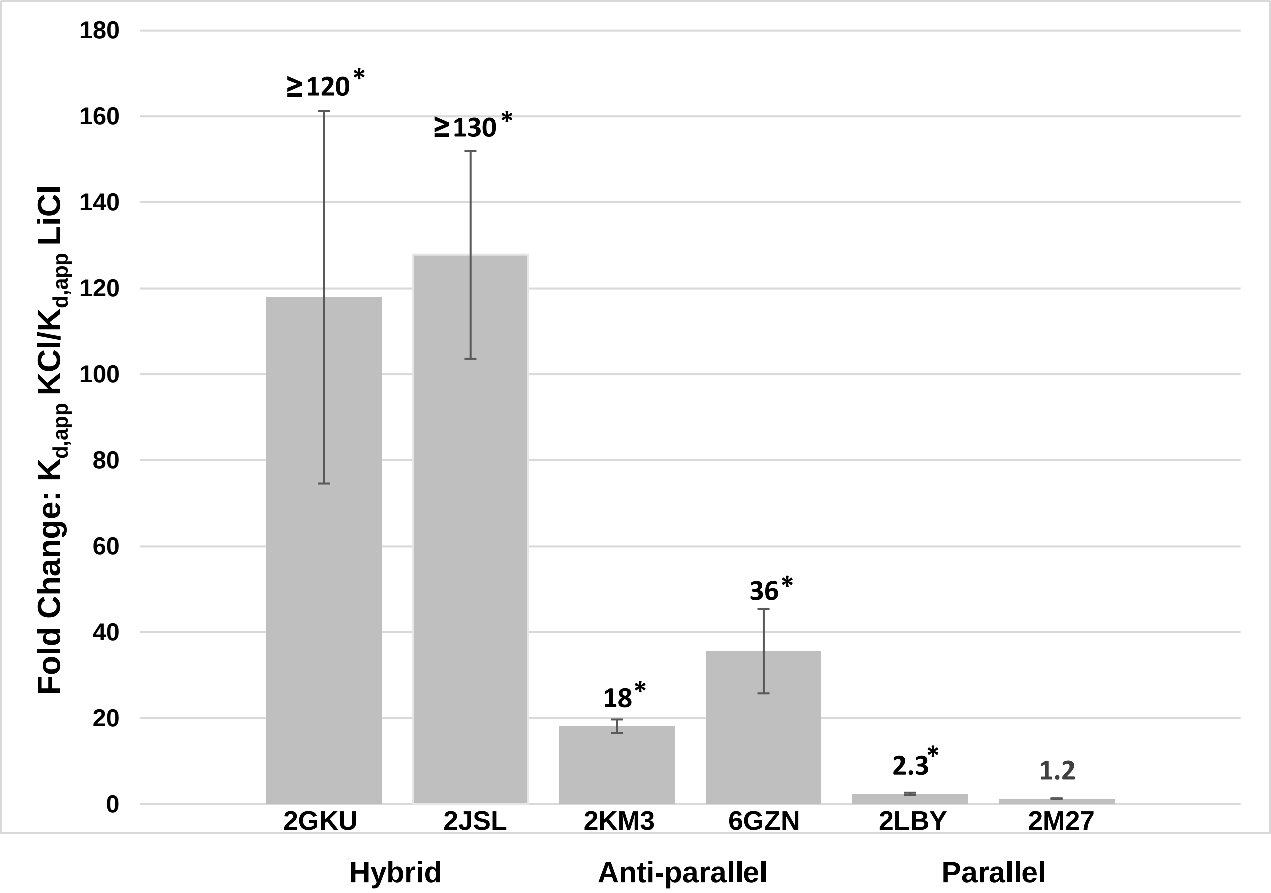

Supplementary Figure S7: CST binding to G4s of hybrid, anti-parallel and parallel topologies. A) Six ligands used for binding with CST, their topologies, length and DNA sequence. B) Fold change of CST binding to each ligand in their linear form (in LiCl) versus the G4 form (in KCl). The larger the fold-change value, the worse CST can bind to the G4 ligand compared to the linear ligand. Error bars represent the standard error of the fold-change. *Indicates the differences in binding affinity between G4 and linear ssDNA is statistically significant with a p-value of ≤ 0.05 as determined by a student's two-tailed t-test.

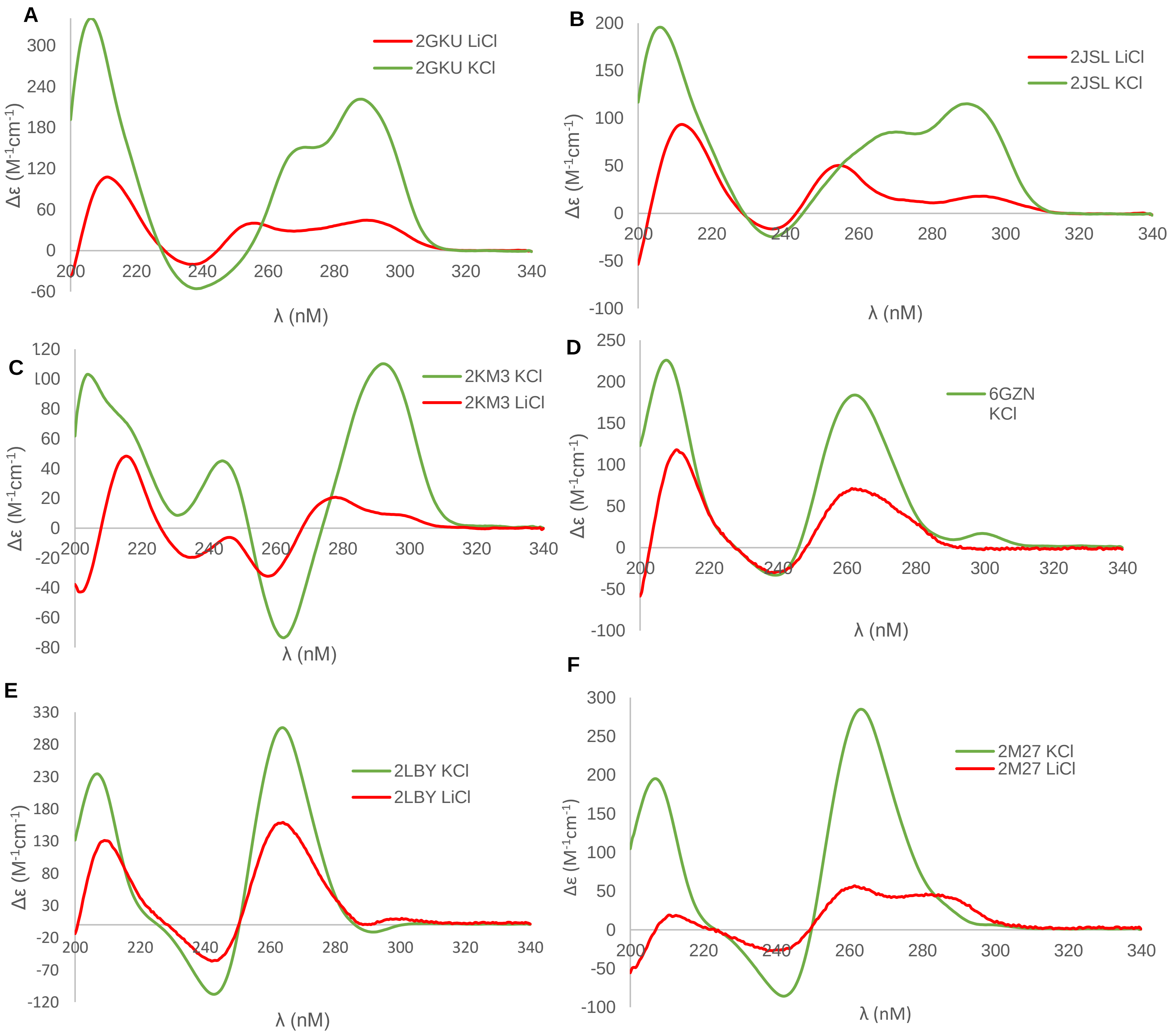

Supplementary Figure S8: Circular dichroism (CD) spectra for G4 topology oligonucleotides. A) CD spectrum of 2GKU confirms hybrid topology in KCl with peaks at ~290 and ~260 nm and a trough at ~240 nm. B) CD spectrum of 2JSL confirms hybrid topology in KCl with peaks at ~290 and ~260 nm and a trough at ~240 nm. C) CD spectrum of 2KM3 confirms anti-parallel topology in KCl with a peak at ~290 nm and a trough at ~260 nm. D) CD spectrum of 6GZN confirms anti-parallel topology in KCl with CD curves matching what was found by Zivkovic et al. (2) which solved 6GZN's structure as anti-parallel. E) CD spectrum of 2LBY confirms parallel topology in KCl with peak at ~260 and a trough at ~240 nm. F) CD spectrum of 2M27 confirms parallel topology in KCl with peak at ~260 and a trough at ~240 nm.

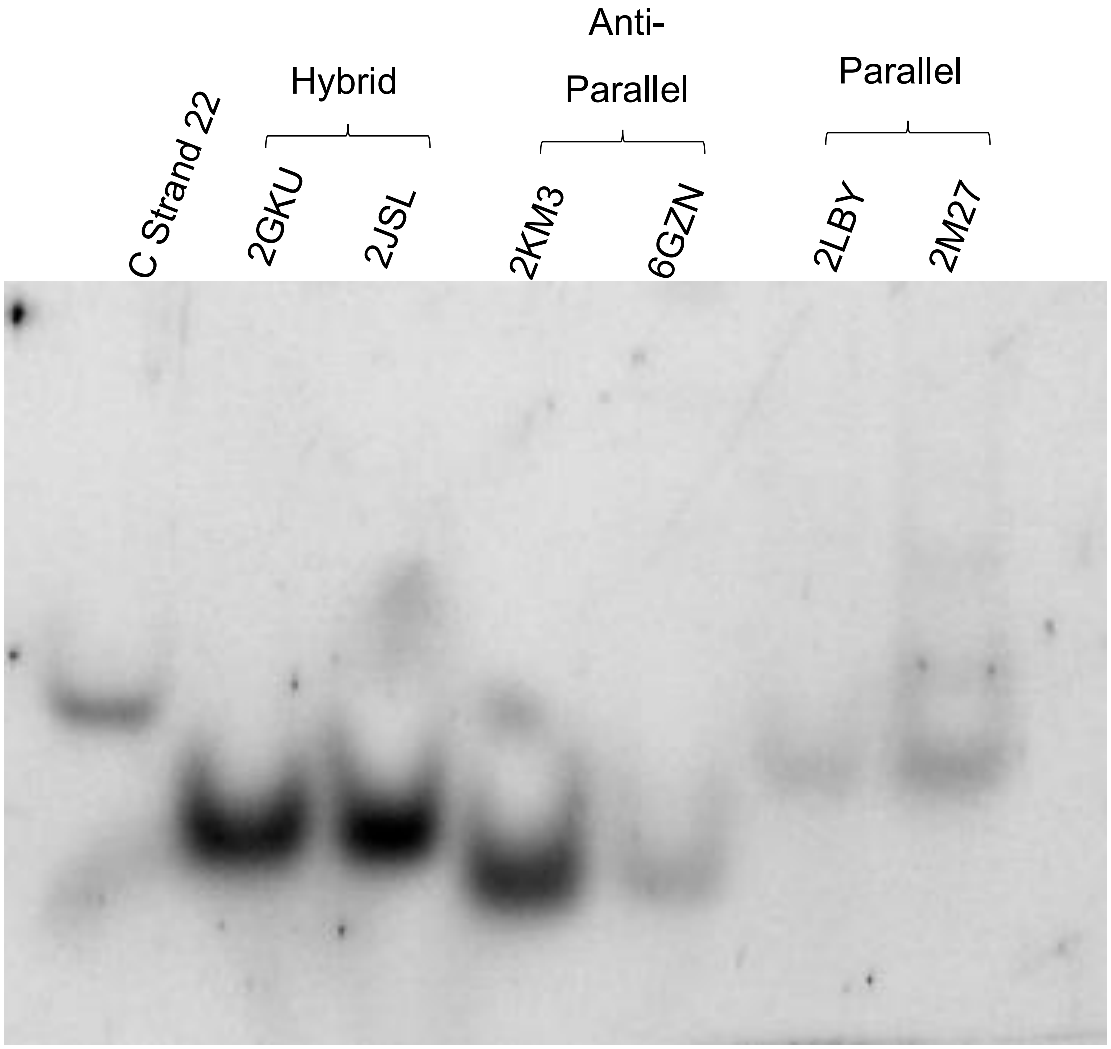

Supplementary Figure S9: Native PAGE gel (0.25xTBE) in KCl (100 mM) for G4 topology oligonucleotides showing G4s are predominantly unimolecular. The single band of the C-strand, hybrid, anti-parallel and 2LBY oligonucleotides shows that the G4 structures are intramolecular and not intermolecular. Multiple faint bands are resolved for 2M27, indicating there may be a mixed population of intra- and intermolecular G4 structures.

A

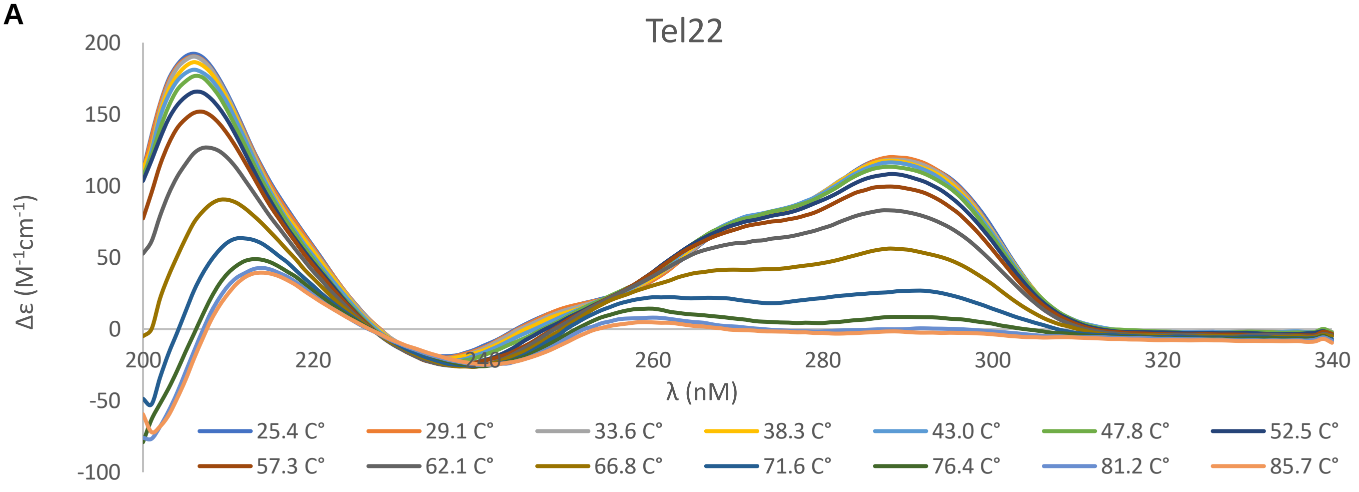

B

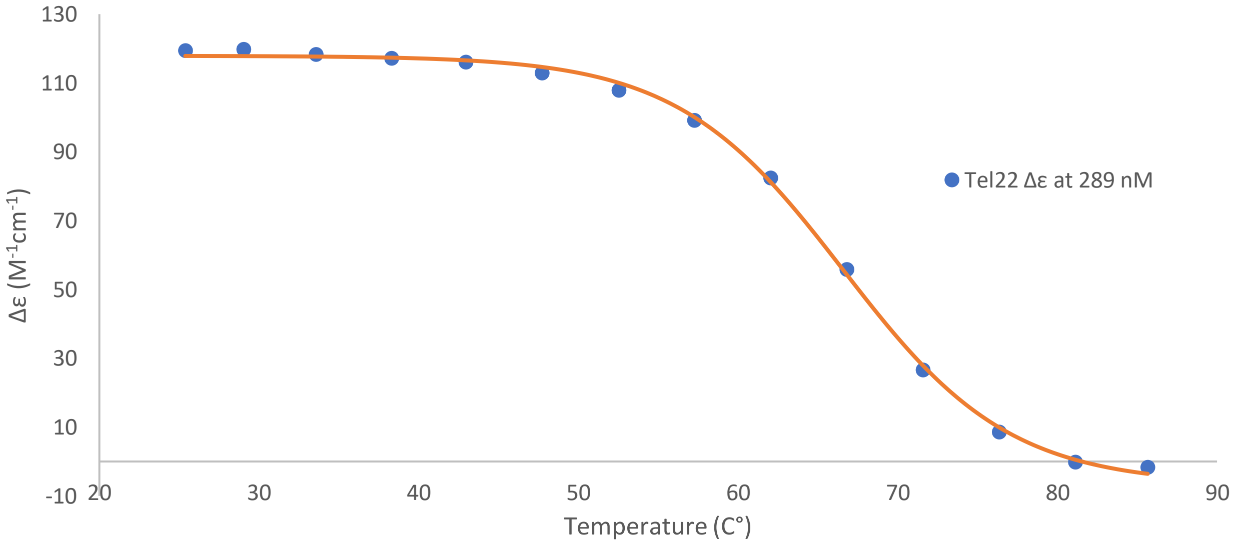

C

**Oligonucleotide**	**T_m (C°)**	**Fold Change CST K_d,app (nM) KCl/LiCl**	**Fold Change RPA K_d,app (nM) KCl/LiCl**
Tel22	66.8 ± 0.2	≥50	5.7
Tel30	65.3 ± 0.2	21	2.5
Tel40	66.6 ± 0.6	1.5	1.2
Tel60	63.8 ± 0.3	7.7	0.86

Supplementary Figure S10: Example melting curve and T_m data for telomere oligonucleotides. A) CD spectra for Tel22 in KCl at increasing temperatures. B) Melting curve for Tel22 plotting the Δε at 289 nM, the peak confirming hybrid topology for Tel22 against the temperature the CD measurement was taken. Data were fit using a Boltzmann sigmoidal curve and T_m's were determined as the halfway point between the upper and lower limits of the sigmoidal curve as described in Materials and Methods. C) T_m data compared to fold change for CST and RPA's binding affinity for the linear versus G4 form for each telomere oligonucleotide. T_m's are an average of *n*=3 melting curves.

A

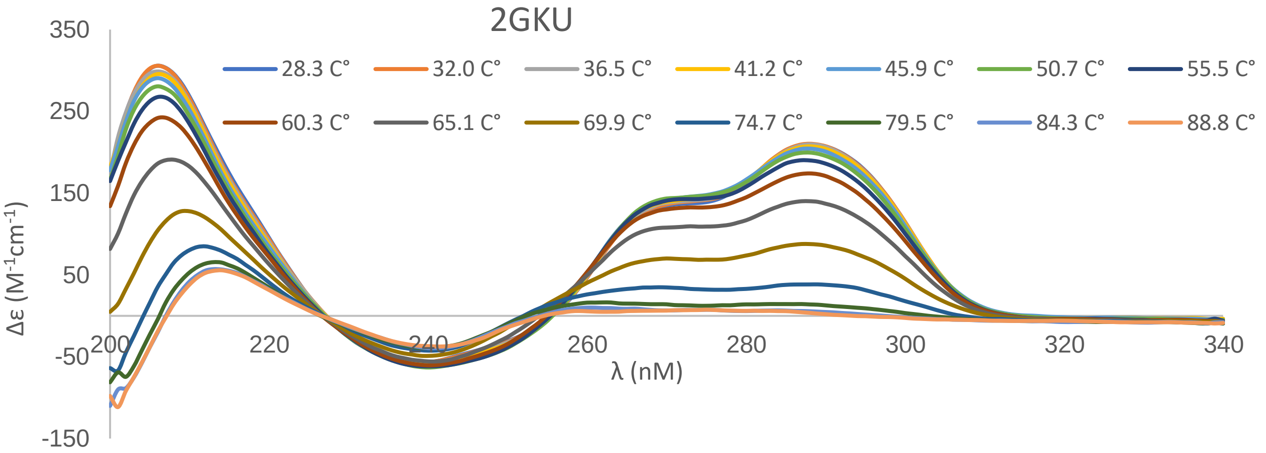

B

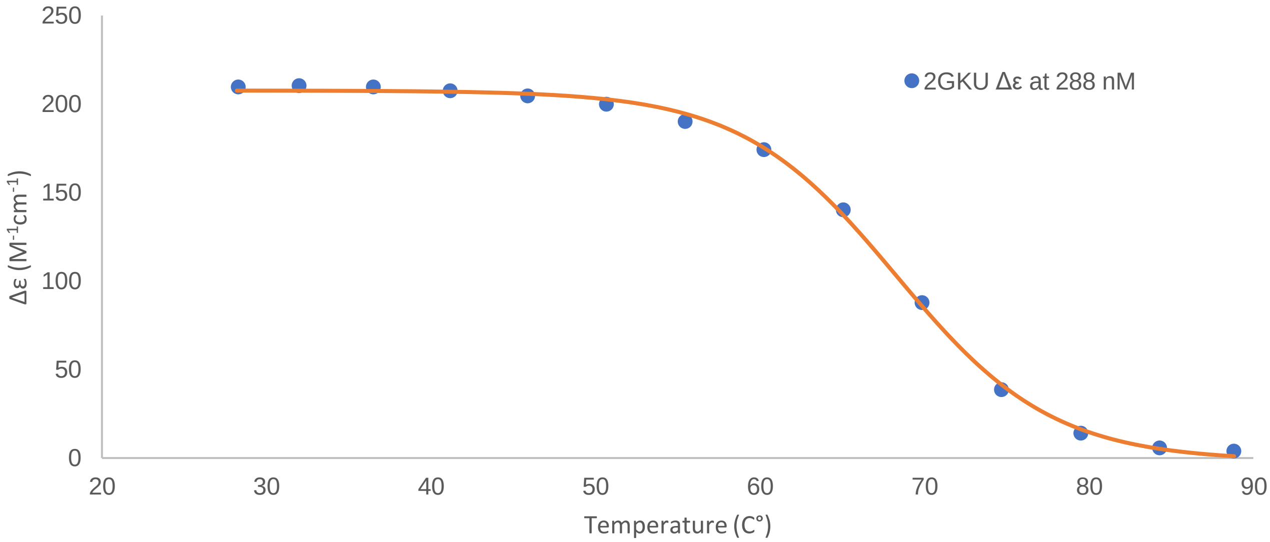

C

Oligonucleotide	T_m (C°)	CST K_d,app (nM) fold change KCl/LiCl
2LBY	≥82 ± 5	≥120
6GZN	68.4 ± 2.1	≥130
2KM3	63.3 ± 1.6	18
2M27	>90	36
2JSL	63.3 ± 1.7	2.3
2GKU	68.8 ± 1.2	1.2

Supplementary Figure S11: Example melting curve and T_m data for G4 topology oligonucleotides. A) CD spectra for 2GKU in KCl at increasing temperatures. B) Melting curve for 2GKU plotting the Δε at 288 nM, the peak confirming hybrid topology for 2GKU against the temperature the CD measurement was taken. Data were fit using a Boltzmann sigmoidal curve and T_m's were determined as the halfway point between the upper and lower limits of the sigmoidal curve as described in Materials and Methods. C) T_m data compared to fold change for CST's binding affinity for the linear versus G4 form for each topology oligonucleotide. T_m's are an average of *n*=3 melting curves.

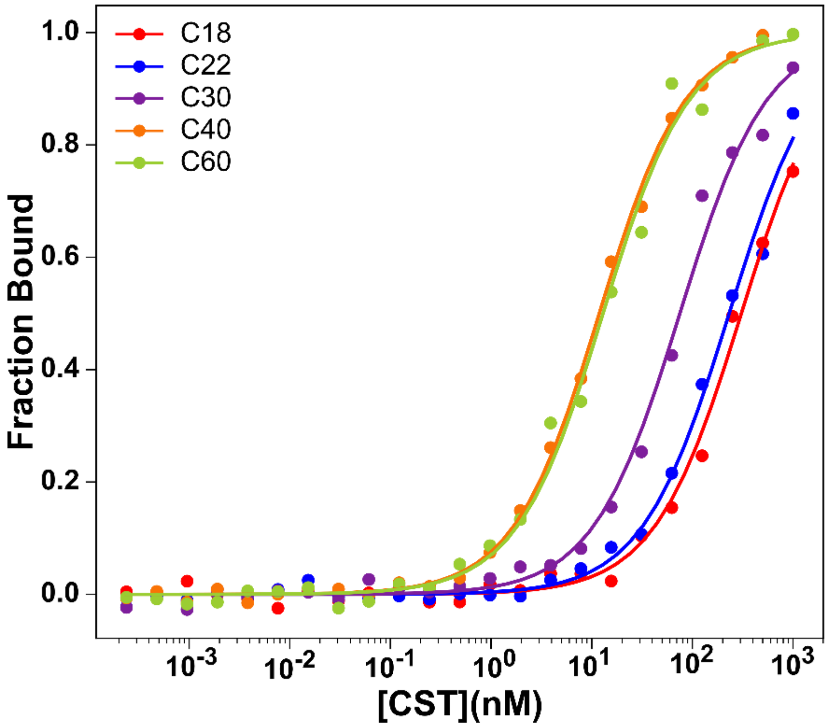

Supplementary Figure S12: Example normalized FA binding curves for CST to C-Strand oligonucleotides showing CST gains affinity as the length of ssDNA increases. Binding done in 200 mM NaCl. For FA binding curves that did not reach completion $K_{d,app}$ values are reported as lower limits for those oligonucleotides.

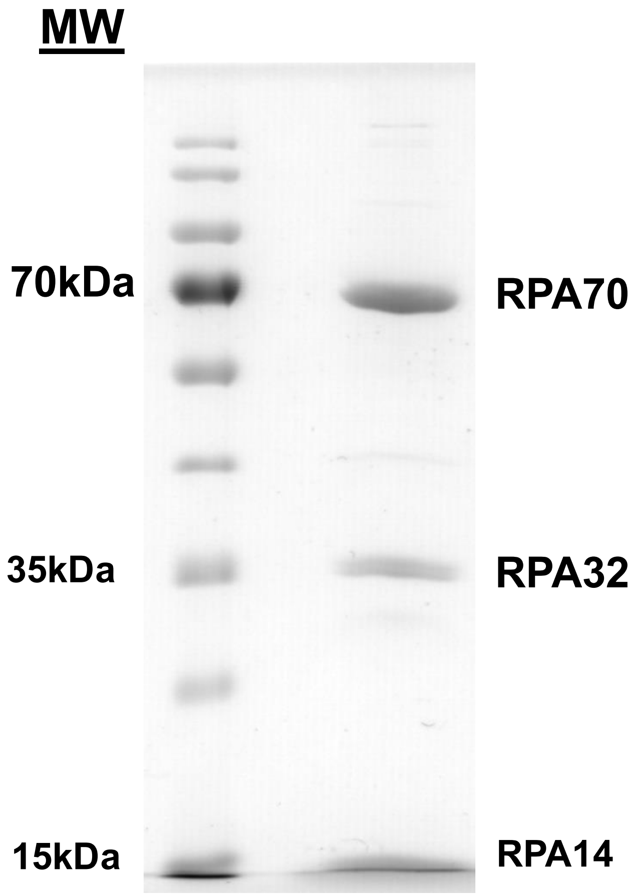

Supplementary Figure S13: RPA purification gel. SDS-PAGE gel for wild-type RPA purification. Purified RPA with each subunit indicated with their corresponding band.

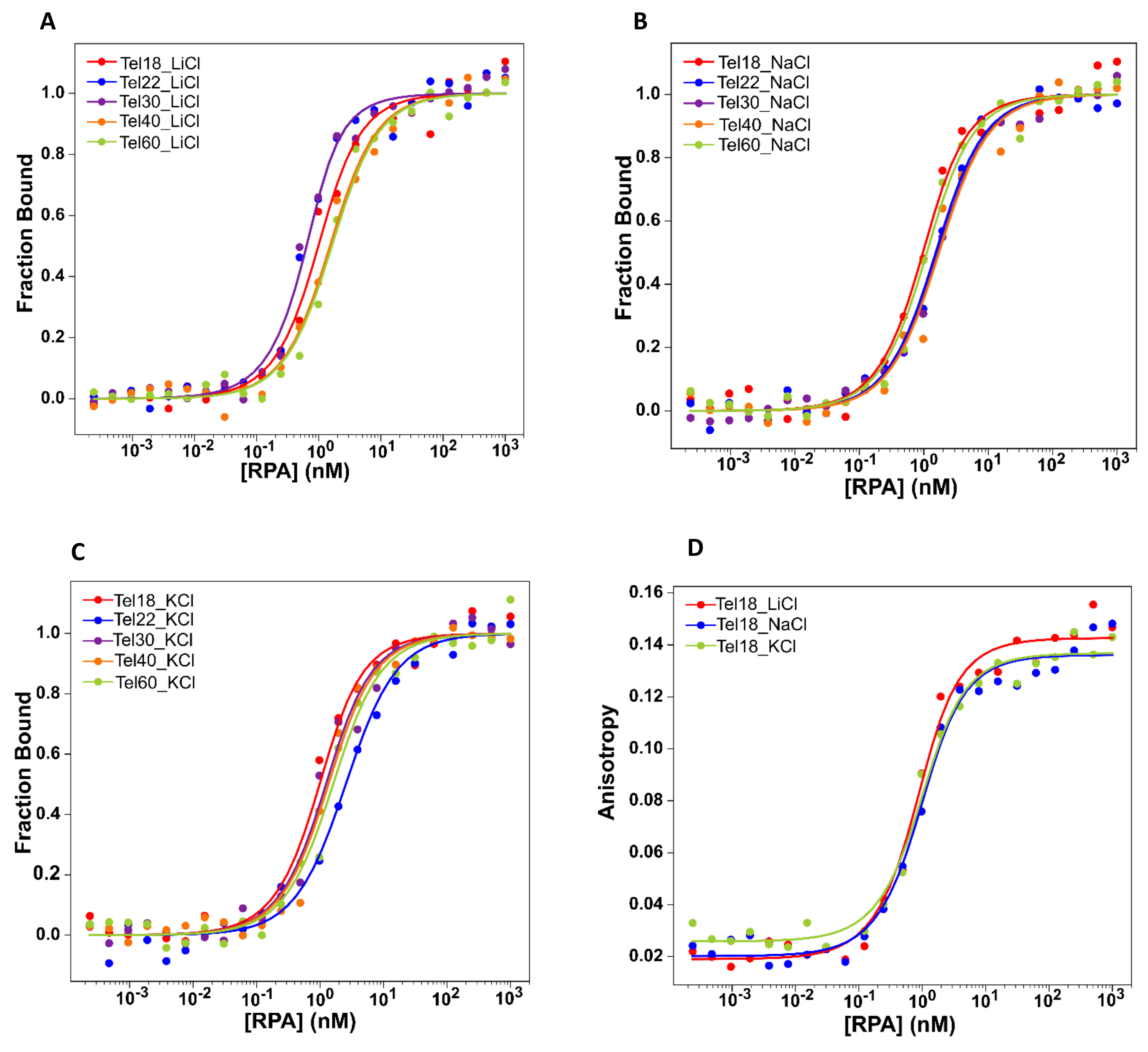

Supplementary Figure S14: (A) Example normalized FA binding curves for RPA to telomere oligonucleotides in 200 mM LiCl. (B) Example normalized FA binding curves for RPA to telomeric oligonucleotides in 200 mM NaCl. (C) Example normalized FA binding curves for RPA to telomeric oligonucleotides in 200 mM KCl. Curves for both A, B and C were fit using equation 1. (D) Representative FA binding curves for RPA to Tel18 showing range of anisotropy values for binding. For FA binding curves that did not reach completion $K_{d,app}$ values are approximations for those oligonucleotides.

A

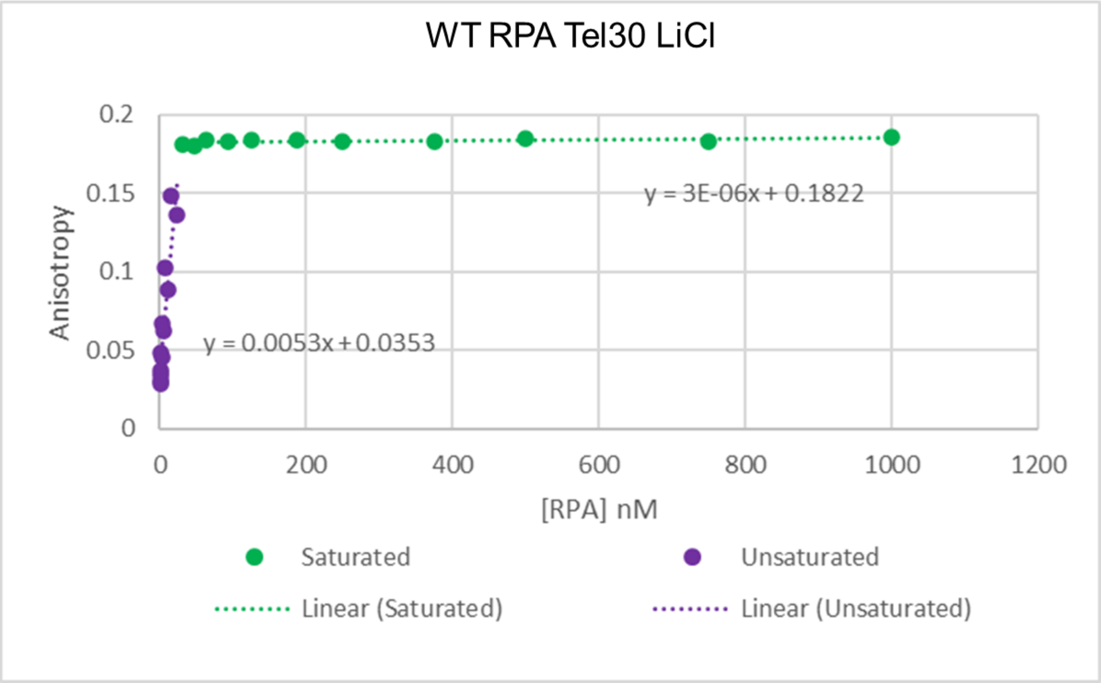

B

	WT RPA	
**DNA Ligand**	**RPA:DNA LiCl**	**RPA:DNA KCl**
Tel22	1.00 ± 0.02	0.95 ± 0.004
Tel30	0.99 ± 0.07	0.96 ± 0.02
Tel40	1.60 ± 0.02	1.56 ± 0.04
Tel60	2.18 ± 0.02	2.15 ± 0.02

Supplementary Figure S15: (A) Example data from WT RPA stoichiometry assay. Ligand used in this experiment is Tel30 in 200 mM LiCl. Concentration of WT RPA on the x-axis and anisotropy values on the y-axis. The purple region is the un-saturated region, and the green is the saturated region. These data points are fit to a linear equation and the intercept of these regions is the protein concentration of RPA needed to bind all of the ligand. (B) RPA stoichiometry of binding to telomeric ligands. RPA binds to Tel22 and Tel30 1:1 in both LiCl and KCl. The approximate 1.5 stoichiometry of RPA to Tel40 is likely due to a mixed population of one or two RPA molecules bound to the DNA. Two molecules of RPA bind to Tel60 in LiCl and KCl. RPA binds to linear and G4 forms of telomeric ligands with the same stoichiometry.

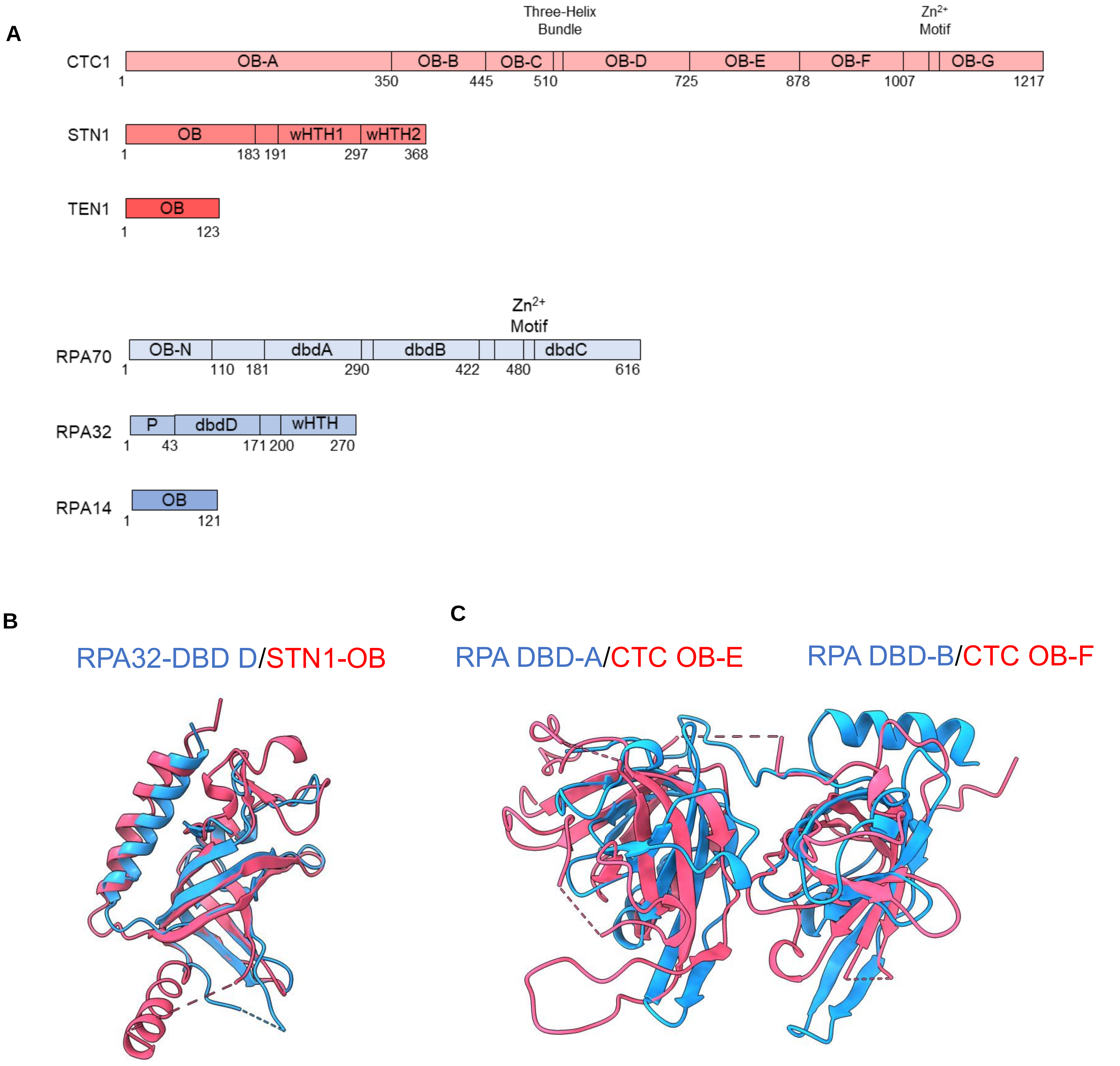

Supplementary Figure S16: (A) Domain maps of hCST (red) (3) and hRPA (blue) (4,5). (B) In blue is the OB fold of RPA32 (PDB 1L1O) (5) and in pink is the OB fold of STN1 (ODB 6W6W) (3) overlayed. (C) In blue is human RPA DBD-A and DBD-B (PDB 1JMC) (6) and in pink is human CST OB-E and OB-F (PDB 6W6W) overlayed. Structures were overlayed and figure generated in UCSF ChimeraX.

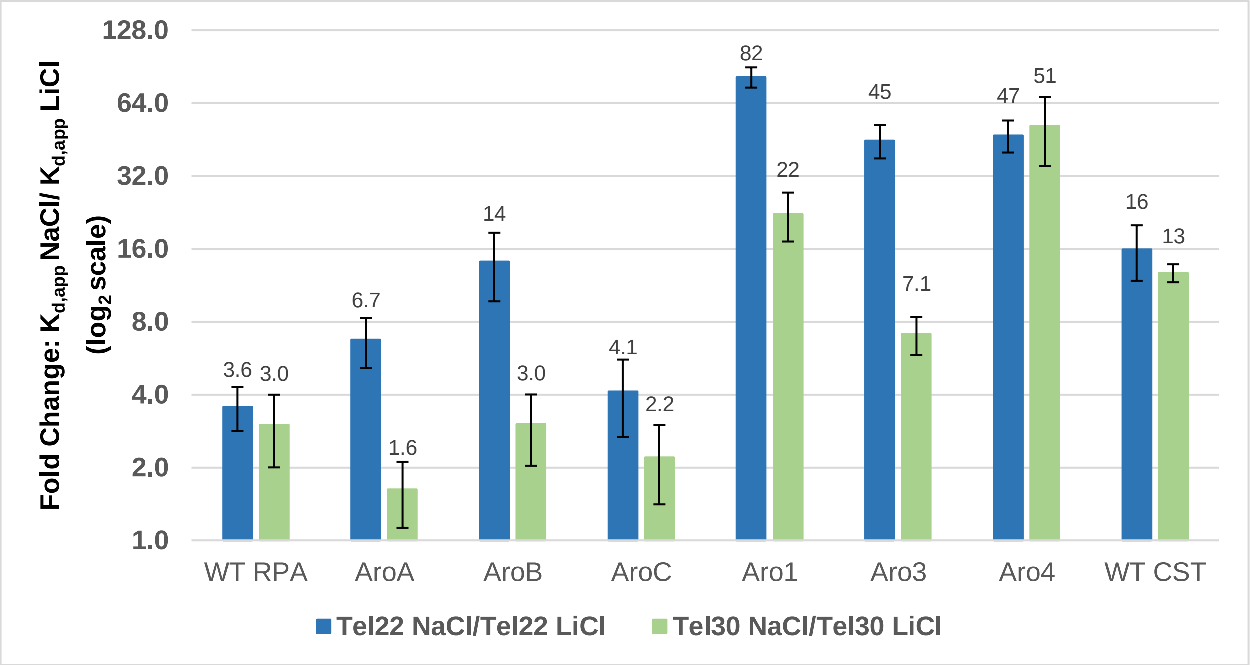

Supplementary Figure S17: Comparison of how telomere G4s affect the binding ability of wild-type CST, wild-type RPA, and RPA mutants. Fold changes for CST, WT RPA, and mutant RPA's to Tel22 LiCl compared to NaCl (blue) and Tel30 LiCl compared to NaCl (green). Fold changes are $K_{d,app}$ NaCl/ $K_{d,app}$ LiCl for each respective protein, where a larger fold change indicates the better the protein binds the oligonucleotide in LiCl than in NaCl. Fold change axis (y-axis) is shown in as log2 (fold change). Error bars represent standard error of fold change. Binding to each oligonucleotide was performed *n*≥ 3 for all proteins in both salts.

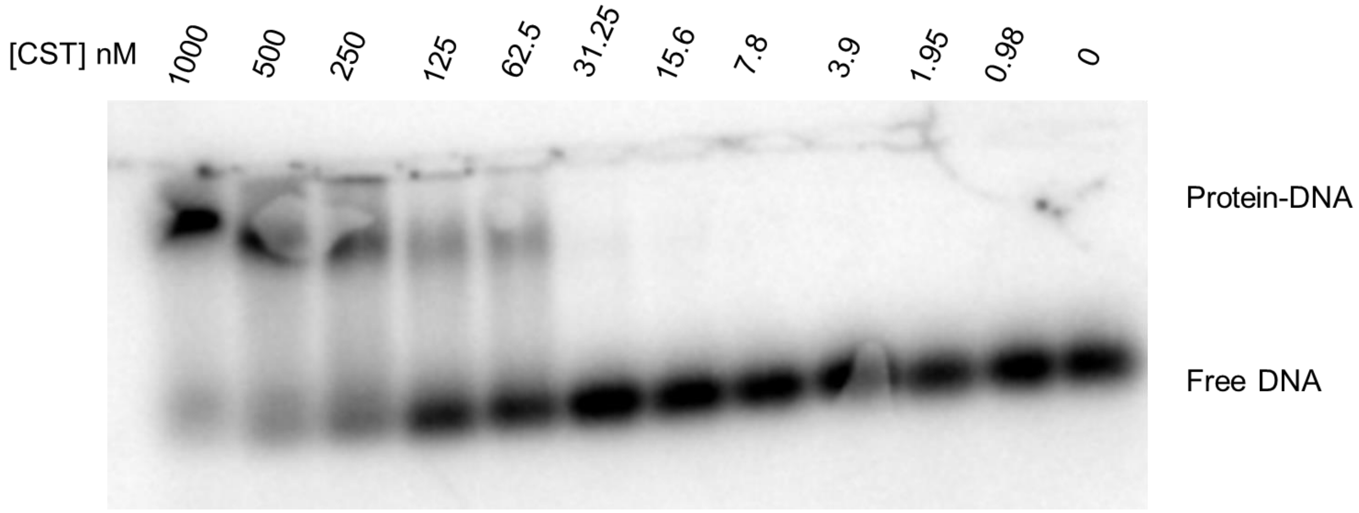

Supplementary Figure S18: Qualitative EMSA showing binding of CST to Tel22 in 200 mM KCl (0.7% agarose, 1X TBE gel). Final concentration of Tel22 is 1nM. Tel22 was heated at 80°C for 10 minutes, then slow cooled to 22-25°C for over 60 minutes. Binding reactions were equilibrated at 22-25°C for 90 minutes before being run on gel (0.7% agarose, 1X TBE) with 1xTBE running buffer containing 100 mM KCl at 70V at 4°C for 60 minutes.

Supplementary Table S1: Sequence and G4 topology for each oligonucleotide used in binding reactions. Each oligonucleotide is also fluorescently tagged on the 5' end with fluorescein group. All oligonucleotides were ordered from IDT.

Oligonucleotide Name	Sequence	Length (nT)	G4 Topology	
			KCl	NaCl
Tel18	$(TTAGGG)_3$	18	hybrid 1+2	anti-parallel
Tel22	$AGGG(TTAGGG)_3$	22	hybrid 1+2	anti-parallel
Tel30	$(TTAGGG)_5$	30	hybrid 1+2	anti-parallel
Tel40	$AGGG(TTAGGG)_6$	40	hybrid 1+2	anti-parallel
Tel40 5' G4	$AGGG(TTAGGG)_3(TGAGTG)_3$	40	hybrid 1+2	anti-parallel
Tel40 Middle G4	$AGTGTGAG(TTAGGG)_4TGAGTGTG$	40	hybrid 1+2	anti-parallel
Tel40 3' G4	$AGTG(TGAGTG)_2(TTAGGG)_4$	40	hybrid 1+2	anti-parallel
Tel60	$(TTAGGG)_{10}$	60	hybrid 1+2	anti-parallel
C18	$(CCCTAA)_3$	18	NA	NA
C22	$(CCCTAA)CCCT_3$	22	NA	NA
C30	$AAT(CCCTAAA)_4CCC$	30	NA	NA
C40	$T(CCCTAA)_6CCC$	40	NA	NA
C60	$AAT(CCCTAA)_9CCC$	60	NA	NA
2KM3	AGGGCTAGGGCTAGGGCTAGGG	22	anti-parallel	NA
6GZN	GGGTAGGGAGCGGGAGAGGG	20	anti-parallel	NA
2LBY	TAGGGAGGGTAGGGAGGGT	19	parallel	NA
2M27	CGGGGCGGGCCTTGGGCGGGGT	22	parallel	NA
2GKU	TTGGGTTAGGGTTAGGGTTAGGGA	24	hybrid 1	NA
2JSL	TAGGGTTAGGGTTAGGGTTAGGGTT	25	hybrid 2	NA

Supplementary Table S2: Binding affinities for all proteins studied against telomeric oligonucleotides in 200 mM NaCl, KCl and LiCl as determined by FA assay. Equilibrium binding constant values for each protein are given as a mean ± SEM for *n* number of experiments.

		LiCl		NaCl		KCl	
		$K_{d,app}$ ± SEM (nM)	*n*	$K_{d,app}$ ± SEM (nM)	*n*	$K_{d,app}$ ± SEM (nM)	*n*
**CST**	Tel18	6.7 ± 0.4	5	7.7 ± 1.6	6	9.3 ± 2.2	3
	Tel22	6.7 ± 1.3	5	106 ± 17	7	≥340 ± 60	11
	Tel30	5.2 ± 0.4	3	67 ± 3	4	112 ± 15	3
	Tel40	0.73 ± 0.27	3	0.74 ± 0.19	6	1.1 ± 0.13	9
	Tel60	1.2 ± 0.4	5	10.6 ± 1.9	8	9.0 ± 2.1	5
**RPA**	Tel18	0.66 ± 0.14	5	0.73 ± 0.05	3	0.50 ± 0.09	4
	Tel22	0.35 ± 0.05	14	1.23 ± 0.19	6	1.99 ± 0.09	4
	Tel30	0.32 ± 0.06	5	0.96 ± 0.21	6	0.81 ± 0.14	5
	Tel40	0.90 ± 0.19	7	1.3 ± 0.4	4	1.1 ± 0.4	6
	Tel60	0.80 ± 0.26	3	0.79 ± 0.22	4	0.69 ± 0.24	4
**AroA**	Tel18	0.45 ± 0.15	4	0.79 ± 0.12	4	0.34 ± 0.06	3
	Tel22	0.36 ± 0.06	9	2.4 ± 0.4	12	2.5 ± 0.3	7
	Tel30	0.29 ± 0.05	9	0.48 ± 0.12	13	0.77 ± 0.28	4
**AroB**	Tel18	6.7 ± 0.4	5	0.50 ± 0.11	4	0.66 ± 0.18	5
	Tel22	0.53 ± 0.15	5	7.5 ± 1.1	6	6.7 ± 1.9	5
	Tel30	0.36 ± 0.07	4	1.07 ± 0.29	4	1.9 ± 0.3	3
**AroC**	Tel18	0.49 ± 0.16	4	0.59 ± 0.14	6	0.26 ± 0.03	8
	Tel22	0.56 ± 0.16	4	2.3 ± 0.5	4	6.4 ± 1.0	4
	Tel30	0.28 ± 0.09	6	0.62 ± 0.11	5	1.62 ± 0.26	7
**Aro1**	Tel18	5.6 ± 1.4	4	4.7 ± 0.7	3	2.5 ± 0.3	6
	Tel22	3.1 ± 0.9	7	254 ± 24	8	>800	6
	Tel30	0.37 ± 0.04	3	8.2 ± 1.6	11	11.9 ± 2.8	5
**Aro3**	Tel18	0.65 ± 0.08	4	0.24 ± 0.03	4	0.56 ± 0.05	4
	Tel22	0.58 ± 0.07	6	26.1 ± 2.9	5	>2000	4
	Tel30	0.21 ± 0.03	14	1.5 ± 0.4	5	175 ± 30	6
**Aro4**	Tel18	5.1 ± 0.5	4	3.5 ± 0.3	4	9.1 ± 0.3	3
	Tel22	6.9 ± 1.0	4	323 ± 19	5	>5000	4
	Tel30	0.29 ± 0.08	4	15.0 ± 2.3	4	840 ± 90	4

Supplementary Table S3: Binding affinities for CST with G4 oligonucleotides of different topologies. *Indicates the differences in binding affinity between G4 and linear ssDNA is statistically significant with a p-value of ≤ 0.05 as determined by a student's two-tailed t-test.

		WT CST, LiCl		WT CST, KCl		
**Ligand**	**Topology**	**$K_{d,app}$ (nM)**	***n***	**$K_{d,app}$ (nM)**	***n***	**$K_{d,app}$ (nM) fold change KCl/LiCl**
2GKU	Hybrid	11.9 ± 1.2	3	≥ $1.4x10^3$	3	≥ 120 *
2JSL	Hybrid	8.3 ± 0.6	3	≥ $1.1x10^3$	3	≥ 130 *
2KM3	Anti-parallel	4.5 ± 0.3	3	81 ± 4	3	18*
6GZN	Anti-parallel	1.1 ± 0.3	3	39 ± 7	3	36*
2LBY	Parallel	11.5 ± 1.3	3	26.5 ± 1.5	3	2.3*
2M27	Parallel	10.8 ± 0.2	3	13.3 ± 0.9	3	1.2

Supplementary Table S4: Wild-type RPA binding affinity for Tel22 in 200 mM LiCl at different concentrations of Tel22. There was found to be no statistically significant difference between the data sets determined by a two-tailed t-test (p=0.56)

Tel22 Concentration	$K_{d,app}$ ± SEM (nM)	*n*
750 pM	0.38 ± 0.08	7
375 pM	0.32 ± 0.05	7

Supplementary Table S5: Binding affinities for wild-type RPA to telomeric oligonucleotides of 40 nucleotides in length which vary where the G4 forms. Binding done in NaCl, KCl and LiCl. For fold change. *Indicates the differences in binding affinity between G4 and linear ssDNA is statistically significant with a p-value of ≤ 0.05 as determined by a student's two-tailed t-test.

	LiCl		NaCl		KCl			
	$K_{d,app}$ ± SEM (nM)	*n*	$K_{d,app}$ ± SEM (nM)	*n*	$K_{d,app}$ ± SEM (nM)	*n*	Fold Change $K_{d,app}$ NaCl/LiCl	Fold Change $K_{d,app}$ KCl/LiCl
Tel40	0.90 ± 0.19	7	1.3 ± 0.4	4	1.1 ± 0.4	6	1.5	1.2
Tel40 5' G4	0.82 ± 0.15	8	0.78 ± 0.08	4	0.85 ± 0.21	4	0.95	1.0
Tel40 Middle G4	0.61 ± 0.13	8	0.28 ± 0.03	4	0.46 ± 0.05	4	0.45	0.75
Tel40 3' G4	0.68 ± 0.16	8	0.40 ± 0.08	4	0.52 ± 0.11	4	0.59	0.77

Supplementary Table S6: Mutant RPA Binding for telomeric oligonucleotides in X mM NaCl, KCl and LiCl compared to WT RPA. All Fold Changes (FC) are relative to the same telomeric oligonucleotide in the same salt compared to WT RPA ($K_{d,app}$ Mutant RPA/$K_{d,app}$ WT RPA).

		**AroA**	**AroB**	**AroC**	**Aro1**	**Aro3**	**Aro4**
	**Salt**	$\frac{K_{d,app}\ AroA}{K_{d,app} WT}$	$\frac{K_{d,app}\ AroB}{K_{d,app} WT}$	$\frac{K_{d,app}\ AroC}{K_{d,app} WT}$	$\frac{K_{d,app}\ Aro1}{K_{d,app} WT}$	$\frac{K_{d,app}\ Aro3}{K_{d,app} WT}$	$\frac{K_{d,app}\ Aro4}{K_{d,app} WT}$
**Tel18**	NaCl	1.07	0.41	0.80	6.42	0.32	4.75
	KCl	0.68	1.32	0.52	4.95	1.11	18.1
	LiCl	0.55	0.68	0.74	8.49	0.99	7.72
**Tel22**	NaCl	1.97	6.13	1.89	210	21.2	263
	KCl	1.24	3.38	3.21	>400	>1000	>2500
	LiCl	1.04	1.54	1.63	8.98	1.68	19.8
**Tel30**	NaCl	0.50	1.12	0.64	8.5	1.53	15.7
	KCl	0.95	2.32	2.0	14.7	215	1040
	LiCl	0.92	1.11	0.88	1.15	0.65	0.91

REFERENCES

1. Paul,T., Opresko,P.L., Ha,T. and Myong,S. (2022) Vectorial folding of telomere overhang promotes higher accessibility. *Nucleic Acids Res*, **50**, 6271–6283.
2. Lenarčič Živković,M., Rozman,J. and Plavec,J. (2018) Adenine-Driven Structural Switch from a Two- to Three-Quartet DNA G-Quadruplex. *Angew Chem Int Ed Engl*, **57**, 15395–15399.
3. Lim,C.J., Barbour,A.T., Zaug,A.J., Goodrich,K.J., McKay,A.E., Wuttke,D.S. and Cech,T.R. (2020) The structure of human CST reveals a decameric assembly bound to telomeric DNA. *Science*, **368**, 1081–1085.
4. Bochkareva,E., Belegu,V., Korolev,S. and Bochkarev,A. (2001) Structure of the major single-stranded DNA-binding domain of replication protein A suggests a dynamic mechanism for DNA binding. *EMBO J*, **20**, 612–618.
5. Bochkareva,E., Korolev,S., Lees-Miller,S.P. and Bochkarev,A. (2002) Structure of the RPA trimerization core and its role in the multistep DNA-binding mechanism of RPA. *EMBO J*, **21**, 1855–1863.
6. Bochkarev,A., Pfuetzner,R.A., Edwards,A.M. and Frappier,L. (1997) Structure of the single-stranded-DNA-binding domain of replication protein A bound to DNA. *Nature*, **385**, 176–181.
